# A Comparison of Low Read Depth QuantSeq 3′ Sequencing to Total RNA-Seq in FUS Mutant Mice

**DOI:** 10.3389/fgene.2020.562445

**Published:** 2020-11-19

**Authors:** Seth Jarvis, Nicol Birsa, Maria Secrier, Pietro Fratta, Vincent Plagnol

**Affiliations:** ^1^UCL Queen Square Institute of Neurology, London, United Kingdom; ^2^UCL Genetics Institute, London, United Kingdom; ^3^UK Dementia Research Institute, University College London, London, United Kingdom

**Keywords:** RNA, ALS, bioinformatics, RNA-Seq, Fused in sarcoma, QuantSeq

## Abstract

Transcriptomics is a developing field with new methods of analysis being produced which may hold advantages in price, accuracy, or information output. QuantSeq is a form of 3′ sequencing produced by Lexogen which aims to obtain similar gene-expression information to RNA-seq with significantly fewer reads, and therefore at a lower cost. QuantSeq is also able to provide information on differential polyadenylation. We applied both QuantSeq at low read depth and total RNA-seq to the same two sets of mouse spinal cord RNAs, each comprised by four controls and four mutants related to the neurodegenerative disease amyotrophic lateral sclerosis. We found substantial differences in which genes were found to be significantly differentially expressed by the two methods. Some of this difference likely due to the difference in number of reads between our QuantSeq and RNA-seq data. Other sources of difference can be explained by the differences in the way the two methods handle genes with different primary transcript lengths and how likely each method is to find a gene to be differentially expressed at different levels of overall gene expression. This work highlights how different methods aiming to assess expression difference can lead to different results.

## Introduction

Amyotrophic lateral sclerosis (ALS) is a progressive neurodegenerative disease with multiple causes. Only 10% of cases have a family history (Kurland and Mulder, 1955; Taylor et al., 2016) with the rest being sporadic. Mutations in genes which encode RNA binding proteins have been associated with the development of ALS. There is therefore increasing interest in how genetic mutations can affect changes in RNA expression and lead to ALS (Boylan, 2015). One of the genes that has been shown to cause ALS is Fused in sarcoma (FUS); mutations in its coding sequence cause FUS, which is usually prevalently nuclear, to mislocalize to the cytoplasm. This mislocalization leads to cytoplasmic inclusions that are the hallmark of disease and further to nuclear depletion of the protein (Kwiatkowski et al., 2009; Vance et al., 2009). Whether the excess of FUS in the cytoplasm or its loss from the nucleus are the drivers of disease remains an open question, and although there is evidence supporting FUS mutations causing gain of function, the loss of nuclear function may also contribute to disease (Scekic-Zahirovic et al., 2016; Birsa et al., 2020).

Alterations in the levels of FUS induce significant changes in splicing and expression (Ishigaki et al., 2012; Coady and Manley, 2015; Humphrey et al., 2020). In order to assess effects of FUS mutations on RNA-expression, we previously generated mouse models that carry mutations in the endogenous *Fus* gene to avoid overexpression artifacts, allowing us to more accurately see any true effects of mutations and separate them from noise caused by FUS overexpression. These mutant mice carry a mutation inducing the skipping of the penultimate exon 14, and will henceforth be referred to as d14 mice (Devoy et al., 2017). In order to address whether the differences caused by FUS mutations differ from the loss of FUS, we have previously conducted total RNA sequencing in parallel on a set of FUS knockout (FUS KO) and control mice and FUS mutants (FUS d14) with their respective controls, and found the expression changes induced by the mutations are mostly due to loss of function (Humphrey et al., 2020).

We have now also sequenced the same samples using a form of 3′ sequencing called QuantSeq to compare the performance of these two different approaches in evaluating gene expression. QuantSeq aims to be able to provide useful information about differential expression at lower read depths than other methods, as well as providing data on differential polyadenylation.

In order to investigate the viability of using QuantSeq as a possible replacement for total RNA-seq, we compared the results of the sequencing of two datasets; FUS KO and FUS d14, each against their own wild-type (WT) littermate controls. We observed significant differences between the genes that are found to be differentially expressed using the two methods. While some of the differences will have come from the fact that QuantSeq uses only mRNA and total RNA-seq uses all RNA-present in the cell, we have identified some other possible causes of the differences in which genes are recognized as differentially expressed, including differences in how the two methods handle genes based on the number of reads they have and the length of their primary transcript.

## Methods

### Preparation of Mouse Models

Fus d14 models were created as previously described (Devoy et al., 2017). Fus knockout mice were obtained from the Mouse Knockout Project [FUStm1(KOMP)Vlcg]. All procedures for the care and treatment of animals were in accordance with the Animals (Scientific Procedures) Act 1986 Amendment Regulations 2012.

### RNA Sequencing

For RNA sequencing experiments Fus d14 or KO heterozygous and homozygous mice were compared to their respective WT littermates. Spinal cords were collected from E17.5 mouse embryos. Tissues were snap frozen, genotyped and total RNA was extracted from the appropriate samples using Qiazol followed by the mini RNAeasy kit (Qiagen). RNA samples used for sequencing all had RIN values of 9.9 or 10. The same samples were used for total and QuantSeq sequencing. For total RNA-seq, cDNA libraries were made at the Oxford Genomics facility using a TruSeq stranded total RNA RiboZero protocol (Illumina). Libraries were sequenced on an Illumina HiSeq to generate paired end 150 bp reads. For QuantSeq libraries the 3′ mRNA-seq library prep kit REV for Illumina (Lexogen) was used QuantSeq and samples were sequenced by the Lexogen facility (Austria). In QuantSeq the average number of reads was 933,955 in d14 and 1,453,108 in KO. In RNA-seq the average number of reads was 35,678,902 in d14 and 39,159,292 in KO.

### Alignment

Alignment of the RNA-seq samples was performed using an in house pipeline. It trims the samples using trim galore (Krueger, 2016), sequences using STAR (Dobin et al., 2013), then uses HTSeq (Anders et al., 2015) to get the per exon counts which are added together to get the per gene counts. To align our QuantSeq samples we used a pipeline created by Gregor Rot designed for 3′ end sequencing. The description of this pipeline can be found in Rot et al. (2017).

### Differential Expression

The per gene counts for both RNA-seq and QuantSeq data were imported to R. Differential expression analysis was then performed using DESeq2 (Love et al., 2014). These results were then used to perform the analysis for our dataset in combination with information from the polyA atlas (Gruber et al., 2016). *Z*-scores were calculated as a derivative of the *p*-value. Some analysis was done using the R packages Enrichment Browser (Geistlinger et al., 2016) and limma (Ritchie et al., 2015), as well as extensively using the tidyverse. All code used in order to perform the analysis can be found here: https://github.com/SethMagnusJarvis/QuantSeqComparison. The number of genes that are differentially expressed differ slightly from our previously published data (Humphrey et al., 2020) because we use a cut-off for lowly expressed genes that was not included our previous analysis. Sampling of our RNA-seq data was performed by producing a list with a gene name in the list once for each read in our initial dataset, then using the sample function in R to get random rows in this list a certain number of times, the number of times each name was found was then counted and used as the new read list.

## Results

### Preparation and Information on Samples

**FIGURE 1:**
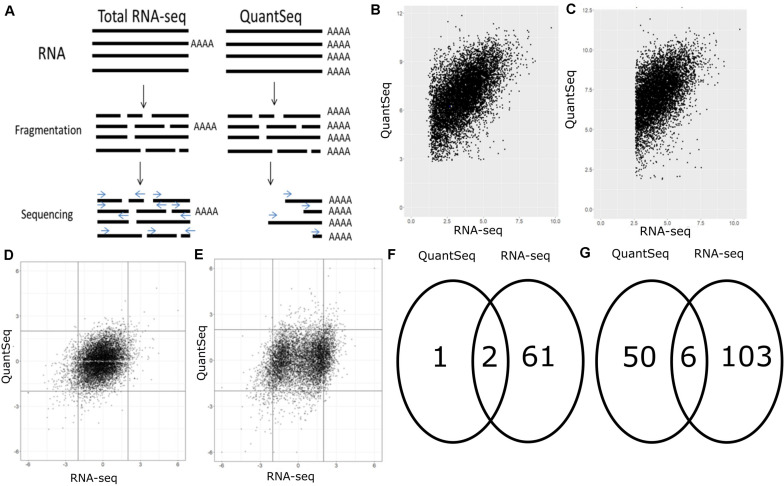
Core differences between QuantSeq and RNA-seq. **(A)** Comparison of the methods of QuantSeq and total RNA-seq; **(B,C)** gene by gene plot comparing log of the BaseMean from DESeq2 between RNA-seq and QuantSeq in **(B)** d14 and **(C)** KO datasets; **(D,E)** comparison of *Z*-scores (a normalized version of the unadjusted *p*-value) in QuantSeq and RNA-seq in **(D)** d14 and **(E)** KO datasets; **(F,G)** Venn diagrams showing overlap of significant genes (padj < 0.05) between QuantSeq and RNA-seq in **(D)** d14 and **(E)** KO datasets.

We performed our methods comparison on two sets of samples derived from embryonic spinal cords of: (a) four Fus KO mice and WT littermate controls, and (b) four Fus d14 mice with their own littermate WT controls. The same RNA samples were then sequenced either using standard total RNA-seq for library preparation or QuantSeq kits produced by Lexogen. QuantSeq selectively amplifies regions of RNA close to a polyA signal, whilst total RNA-seq sequences all pieces of RNA present within the cell regardless of a presence of a polyA signal. The core differences between the two methods are illustrated by Figure 1A.

### QuantSeq and RNA-Seq Have Similar Levels of Reads PER Gene

We investigated how expression of single genes (Figures 1B,C) compared between methods and found that the BaseMean value correlated in both d14 and KO samples (cor = 0.3515, *p* < 0.0005 in the d14 datasets, and cor = 0.3586, *p* < 0.0005 in the KO datasets). This means that the two methods see roughly the same number of reads after normalization overall in each gene and that any differences observed are due to other factors.

### QuantSeq and RNA-Seq Only Have a Moderate Overlap in Significantly Differentially Expressed Genes

Our RNA-seq pipeline found several pseudogenes and genes which had not been experimentally confirmed to be significant. These were removed. We then assessed whether the differential expression between each mutant and its control group was similar using the two sequencing methods. The *Z*-scores do show that changes do occur, with rare exceptions, in the same direction, and show a weak correlation in both datasets (Figures 1D,E). This was especially true for genes where the absolute *Z*-score was >2, the genes that are most likely to be significant. A comparison of genes which were significant at an adjusted level (padj < 0.05) (Figures 1F,G) showed that in our d14 dataset (Figure 1F), only 2 of the 70 genes that RNA-seq found to be significantly differentially expressed, were also found to be significantly differentially expressed in QuantSeq. These genes are *Pspc1* and *Selenop*. QuantSeq also found one gene – *Mt2* – to be significantly differentially expressed that was not observed in RNA-seq. The percentage of significant genes which overlap in KO (Figure 1G) is even smaller. Only about 10% of the genes found to be significantly differentially expressed in QuantSeq were also found significant by RNA-seq. The genes that were found to be significantly differentially expressed *Trim72*, *Fus*, *Bcas1*, *Gjd2*, *Ahi1*, and *Chodl* are linked to, among other things, cell repair and development of the nervous system.

Since the overlap in which genes were found to be significant was poor, we decided to compare unadjusted *p*-values in one dataset to adjusted *p*-values in the other (Supplementary Figure 1) in order to determine whether the issue was genes that showed some significance, but missed the threshold for adjusted significance possibly due to poor read depth. In the KO data we found that when we relaxed the QuantSeq threshold, the percentage of RNA-seq genes that overlapped rose from 5 to 10%. There was a bigger increase when we relaxed the RNA-seq threshold, where the overlap with QuantSeq rose from 10 to 36% of the total genes QuantSeq found to be significant.

To further investigate the discrepancy observed between the differentially expressed genes from the RNA-seq and QuantSeq experiments, we compared the rank of gene expression in one dataset to the rank in the other. As expected, we found a positive correlation between techniques, and interestingly transcripts found to be significantly changed by only one technique, were also detected at higher level using that same technique, further supporting read depth and coverage as a major source of the discrepancies (Supplementary Figure 2). Ten genes significantly differentially expressed in QuantSeq were not present in the RNA-seq differential expression dataset. Of those, eight had been classified as fusion-gene candidates detected from split reads by our RNA-seq pipeline and two had been filtered out due to having very few reads. The majority of genes that were significantly differentially expressed in the RNA-seq dataset were not found in the differential expression results of our QuantSeq dataset. Supplementary Figure 3 shows the division of these genes. The majority of which had been filtered out because they had too low expression or no expression. Of those which were not found, most were antisense or long non-coding RNAs.

In order to demonstrate that the methods see what we would expect biologically, we plotted the *Z*-scores in KO against those in d14. While RNA-seq did have a more pronounced correlation, and was more likely to find genes to be more highly differentially expressed in KO than d14, for the most part a similar correlation was seen in QuantSeq (Supplementary Figure 4).

We then compared the size of the detected differential expression between methods and found that RNA-seq both tended to show larger fold change in differential expression in the genes that were differentially expressed, and tended have a smaller *p*-value (Supplementary Figures 5–8).

### GO Terms Using QuantSeq and RNA-Seq Differentially Expressed Genes Show a Moderate Overlap

We compared the biological process GO terms arising from the different analyses using genes which were found to be significant with an unadjusted *p*-value < 0.05. This allowed us to have a broader base of GO-terms to potentially see any overlap. Table 1 shows the results of this comparison. The majority of GO terms in both datasets did not overlap, however we could see, especially in the KO dataset (where more GO-terms were found to be significant) that there was a 25% overlap of the total number of GO terms found to be significant. These included GO terms related to sensory perception, localization, transport, and RNA-metabolic processes. This tells us that while the two datasets do differ in which genes they find significant, the processes that those genes serve do overlap to an extent. The full results of which GO terms were found to be significant can be found in Supplementary Tables 1, 2.

**TABLE 1:** Number of significant GO terms and how they overlap in each dataset.

	GO terms only significant in total RNA-seq	GO terms only significant in QuantSeq	GO terms significant in both
d14	28	12	5
KO	186	109	98

### RNA-Seq Finds Fewer Genes When Downsampled to Have the Same Number of Reads as QuantSeq

As stated in the methods, RNA-seq has about 30 times the average number of reads per sample as QuantSeq. We used the sample function in R to take random reads from our RNA-seq dataset, at several different levels between 1.5 million reads and 20 million reads per sample to see how they compared. There was a positive correlation overall, with more reads resulting in more genes found to be differentially expressed. The results in KO can be observed in Supplementary Figure 9. At these lower levels of sampling, QuantSeq does find more genes to be differentially expressed than RNA-seq in KO aside from at 20 million reads per sample and finds as many/more genes differentially expressed below 10 million reads per sample in the d14 dataset.

Whilst the number of detected differentially expressed genes may depend mostly on the number of reads as shown above, our investigation progressed to investigate reasons for the differences between genes that were differentially expressed observed between the two methods. RNA-seq finds about 5× the genes that QuantSeq does represented in the top 10% of reads, and about double the number of genes in the top 50% of reads (Table 2). There are about half the number of genes with reads in QuantSeq compared with RNA-seq meaning by the time 50% of reads have been accounted for, the proportion of total genes represented is similar. This seems to suggest that RNA-seq tends to distribute reads more evenly across genes whereas QuantSeq has a large number of reads concentrated in relatively few genes.

**TABLE 2:** Minimum number of genes required to cover 10 and 50% of total reads in all datasets.

	d14 QuantSeq	d14 RNA-seq	KO QuantSeq	KO RNA-seq
First 10%	15	61	16	64
First 50%	698	1159	646	1224

### Expression Levels and Transcript Length May Contribute to the Differences in Differential Expression Detection

**FIGURE 2:**
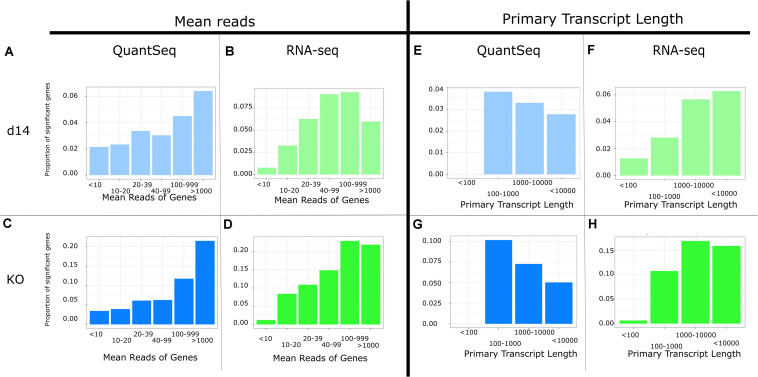
Possible sources of difference between QuantSeq and RNA-seq using d14. **(A,C,E,G)** and KO **(B,D,F,H)** datasets: **(A–D)** bar plots showing the proportion of genes that are significantly differentially expressed (*p*-value < 0.05) separated by the mean number of reads in the gene using **(A,C)** QuantSeq, and **(B,D)** RNA-seq sequencing; **(E–H)** bar plots showing the proportion of genes that are significantly differentially expressed (*p*-value < 0.05) separated by the length of the Appris Primary 1 transcript in the gene using the **(E,G)** QuantSeq, and **(F,H)** RNA-seq sequencing.

We then considered whether features of transcripts can impact on the analysis. We first asked whether the two methods performed differently on high or lowly expressed genes, and we compared proportions of genes that are significantly differentially expressed (*p*-value < 0.05) based on the number of reads, as shown in Figures 2A,B,E,F. We observed a positive correlation between the number of reads and the likelihood that a gene was found to be significantly differentially expressed for both types of library preparation. Total RNA-seq’s correlation plateaued at ∼100 reads while QuantSeq’s did not plateau, suggesting QuantSeq may be less able to detect changes in expression in lowly expressed genes compared to RNA-seq. When using adjusted *p*-values, the correlation in QuantSeq remains, but and RNA-seq is still no more likely to find a gene to be significant over a certain number of reads (Supplementary Figures 10A–D).

We then asked whether the length of the primary transcript impacted on the likelihood of a gene being found significant (Figures 2C,D,G,H). There is a strong positive correlation in RNA-seq, and a strong negative correlation in our QuantSeq datasets suggesting that part of the differences we observe may be linked to how the two methods handle genes with different primary transcript lengths. The negative correlation persists when using adjusted *p*-values in QuantSeq (although all significant genes are concentrated within two groups), but there is no longer obvious correlation in RNA-seq (Supplementary Figures 10E–H).

Finally, as QuantSeq sequences specifically the polyA region, we asked whether either of the two methods found a bias in how likely a gene was to be differentially expressed depending on how many polyA signals each gene had. When we compared the number of polyA signals to the proportion of genes that were significantly differentially expressed (Supplementary Figure 11) there was no correlation in any of the QuantSeq data or in the RNA-seq KO dataset (A–C), and an unexplained negative correlation in RNA-seq (D), this negative correlation disappears when using adjusted *p*-values (Supplementary Figure 12).

### The Differences Are Not Due to the Method of Differential Expression Used

We also looked at how *Z*-scores changed when using different methods of calculating differential expression, specifically we used the R packages limma and edgeR as well as DESeq2 (Supplementary Figures 13, 14). We also compared how the *Z*-scores of the top 25% of genes looked (Supplementary Figure S15), and the *Z*-scores compared in genes with only a polyA signal in the polyA atlas (Supplementary Figure 16). None of these tests showed substantial differences between the methods.

## Discussion

RNA-sequencing is a rapidly evolving form of technology. As such there are often new methods developed. It is important to compare these methods to existing methods to evaluate their relative performance. This is especially true in cases where one method seems to offer substantial advantages in one area. In this case, QuantSeq claims to obtain similar results to total RNA-seq at far lower read depths, as well as providing easily accessible information on differential polyadenylation. Our read depth in QuantSeq is about 30× lower than our depth in RNA-seq, and is lower than some recommendations for use of QuantSeq. However, our dataset was sequenced at Lexogen and this read depth was approved by them, so we believe it is a valid use of the method.

We have found differences between the genes that RNA-seq and QuantSeq find to be differentially expressed. This does not necessarily say that any one method is better than another. It is promising that most genes that are significant in one dataset are at least present in the other dataset and are either not significant or too lowly expressed to run meaningful differential expression on them. This suggests that in QuantSeq, a substantial culprit for some differences in the genes which are found to be significantly differentially expressed is the low number of reads in the dataset. This seems to imply that while QuantSeq does identify the same genes as RNA-seq, the depth at which the sequencing was performed in this experiment may not allow QuantSeq to operate optimally.

We had several hypotheses for why the differences we saw in the *Z*-Scores may have come about given how both of the methods of sequencing work. While we have done our best to remove batch effects by ensuring each mutation has a case control and sequencing each mutant dataset with its relevant case control to hopefully reduce noise, different sequencing centers may introduce biases that we are not aware of. Our investigation showed that QuantSeq performs best when a gene has a high number of reads: by contrast, RNA-seq only requires a baseline number of reads in a gene before it finds a gene to be differentially expressed. RNA-seq does not seem to show a substantial bias toward genes with longer primary transcript length contrary to results by others (Ma et al., 2019). There did seem to be an inverse correlation between length of primary transcript and likelihood of a gene to be differentially expressed in QuantSeq. There did not appear to be a particularly strong correlation in any of the other possible sources of difference we tested.

Splicing changes have been shown to impact on polyA selection, but we have previously investigated the overlap between splicing and expression changes in these datasets (Humphrey et al., 2020) and found this to be modest, therefore making the impact of splicing changes on the different sensitivities likely not to be relevant.

When sampling the dataset, RNA-seq did have reads in more genes, this may have led to RNA-seq finding fewer genes to be significant, it is obvious that QuantSeq is more likely to find genes to be significantly differentially expressed at low levels of reads, this is to be expected since QuantSeq only has one read per transcript so can hopefully get more information, but most of the genes which QuantSeq finds to be significant are not found to be significant by RNA-seq at any level so we cannot be sure how many are real hits. Since Illumina recommends upwards of 30 million reads (Illumina Inc, 2017), when measuring at levels of reads from 3× lower than this, to 20× lower than this, the poor performance of RNA-seq is somewhat expected.

A large number of the genes which are differentially expressed exclusively in RNA-seq have been subsequently validated elsewhere. While we have not been able to experimentally validate the QuantSeq genes, some of them, including three of the five most differentially expressed ones in KO (Mcur1, FTSJ1, and GPR17) have been linked to neurodegeneration (Liao et al., 2017; Angelova et al., 2020; Bonfanti et al., 2020), or in the case of GPR17, directly linked to ALS. Many of the rest have not.

It is possible that other methods are better optimized for use with QuantSeq. Corley et al. (2019) found a much stronger correlation in results than our study, although RNA-seq still found more genes to be differentially expressed than QuantSeq. Since the QuantSeq data used in this article has 30× higher read depth than our data, most of the differences observed are likely in part due to this difference in read depth. Combining QuantSeq with a fast method of quantification like salmon may allow for rapid, inexpensive differential analysis for less focused or more exploratory questions than existing methods.

This study does not demonstrate improved performance in measuring differential expression for either method, but highlights substantial differences between the two methods that should be taken into consideration when designing experiments. QuantSeq finds a lot more genes to be differentially expressed with smaller library sizes than RNA-seq does. The fact that it does seem to provide useful information at lower read depth may mean QuantSeq can act as a cost-effective method to sequence large numbers of samples at low read depth to find genes for further investigation. It is especially worthwhile in this situation if differential polyadenylation is an area of interest.

However, it must be borne in mind that most of these genes are not seen to be differentially expressed by RNA-seq, so some of them may be called into question. RNA-seq is a more widely used library preparation method and, while it is possible that the differences between the two methods are either false positives or false negatives on the part of QuantSeq, more standard library preparation may be preferable for general standardization of results since they have a proven track record of finding true differential expression in genes. The low read depth of our QuantSeq data is likely responsible for a large portion of the difference between the datasets, but we have highlighted some of how the two methods do differ in their handling of data.

The goal of this article is not to disprove QuantSeq’s capability of producing useful results. A demonstration of its utility can be found in Oh et al. (2020) which found that the levels of expression of various cytokines correlated with changes in gene expression found using QuantSeq. As with all methods though, we advise care is taken and validation is performed before any conclusions are reached. Studies conducting multiple approaches in parallel, including qPCR and nanostring, will be informative to compare expression techniques.

## Data Availability Statement

The QuantSeq data has been uploaded to the Sequence Read Archive, accession number PRJNA668024. The total RNA-seq data was uploaded to the Gene Expression Omnibus, accession number GSE147288.

## Ethics Statement

The animal study was reviewed and approved by the UCL Institutional Ethical Review Committee.

## Author Contributions

SJ performed the data analysis. NB generated the data. SJ, PF, and VP designed the study. PF and MS assisted with revisions and improving the final manuscript. PF, MS, and VP supervised the project. SJ wrote the manuscript. All authors contributed to the article and approved the submitted version.

## Conflict of Interest

The authors declare that the research was conducted in the absence of any commercial or financial relationships that could be construed as a potential conflict of interest.
