# Supplementary material for: A Comparison of Low Read Depth QuantSeq 3′ Sequencing to Total RNA-Seq in FUS Mutant Mice
Source: Front Genet. 2020 Nov 19;11:562445. doi: 10.3389/fgene.2020.562445 (PMC7717943; doi:10.3389/fgene.2020.562445)
Supplement: Supplementary file 1 [file Data_Sheet_1.docx]

Supplementary Figures


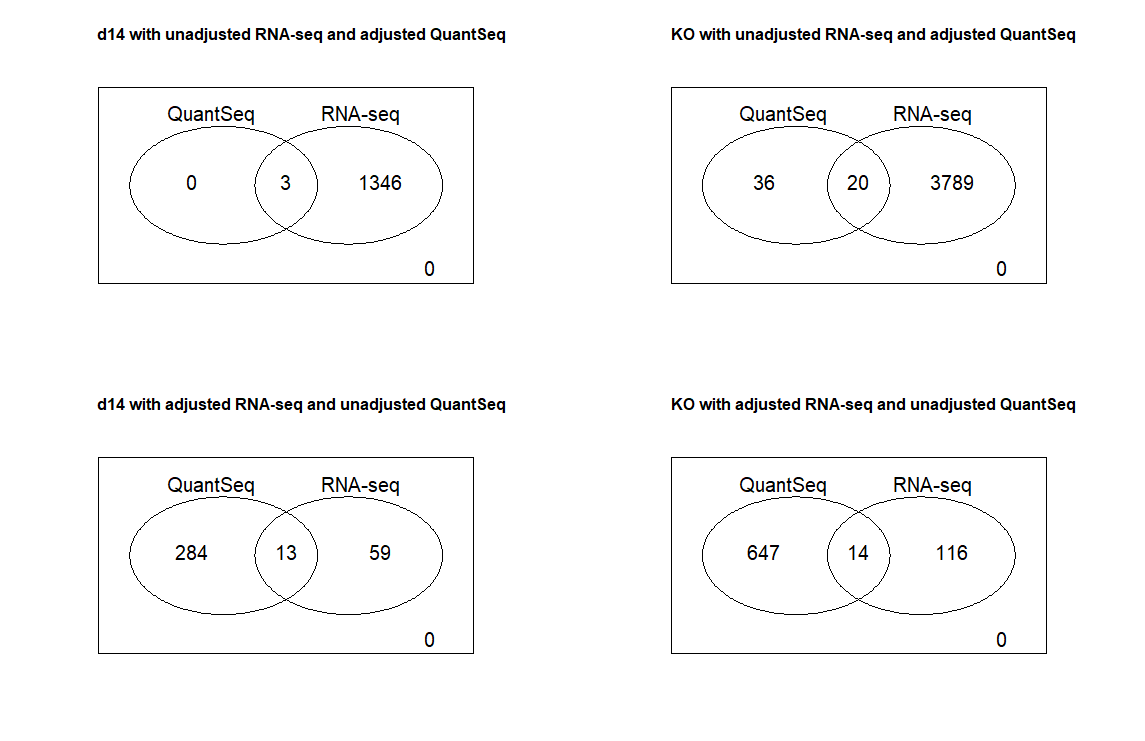


B

D

C

A

Supplementary Figure 1. Venn diagrams showing the comparison genes found significant using an unadjusted p-value of 0.05 in one dataset and an adjusted p-value of 0.05 in the other dataset, using unadjusted RNA-seq p-values in A-B and unadjusted QuantSeq p-values in C-D


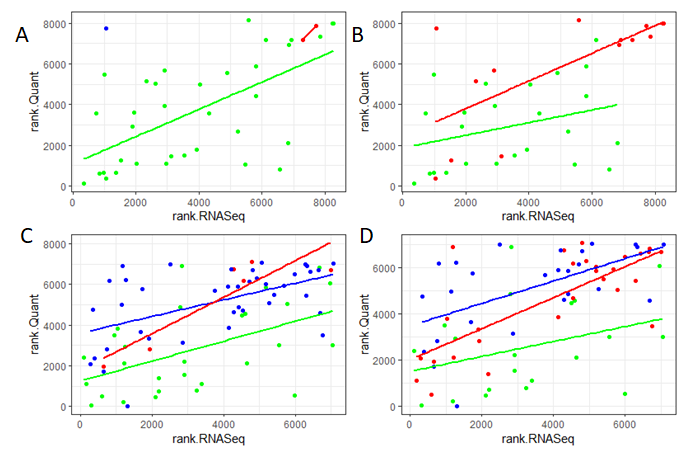


Supplementary Figure 2. Comparison of genes found to be significant in one dataset, ranked by their relative expression in d14 (A-B) and KO (C-D). Genes are coloured green if only significant (padj < 0.05) in RNA-seq, blue if only significant in QuantSeq, and red if significant in both. Panels B &D use a relaxed threshold for red, where if a gene is adjusted significant after multiple testing correction in one dataset, it only needs to have an unadjusted p-value threshold of 0.05 in the other.


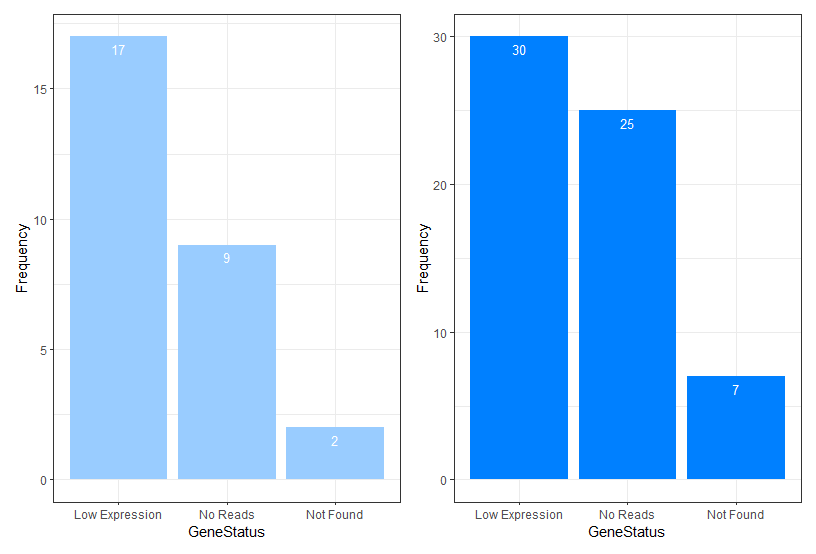


Supplementary Figure 3. Bar plots showing the distribution of genes that were found significant in RNA-seq but were not present in our differential expression dataset of Quantseq after filtering pseudogenes and genes to be experimentally confirmed in (A) d14 and (B) KO.


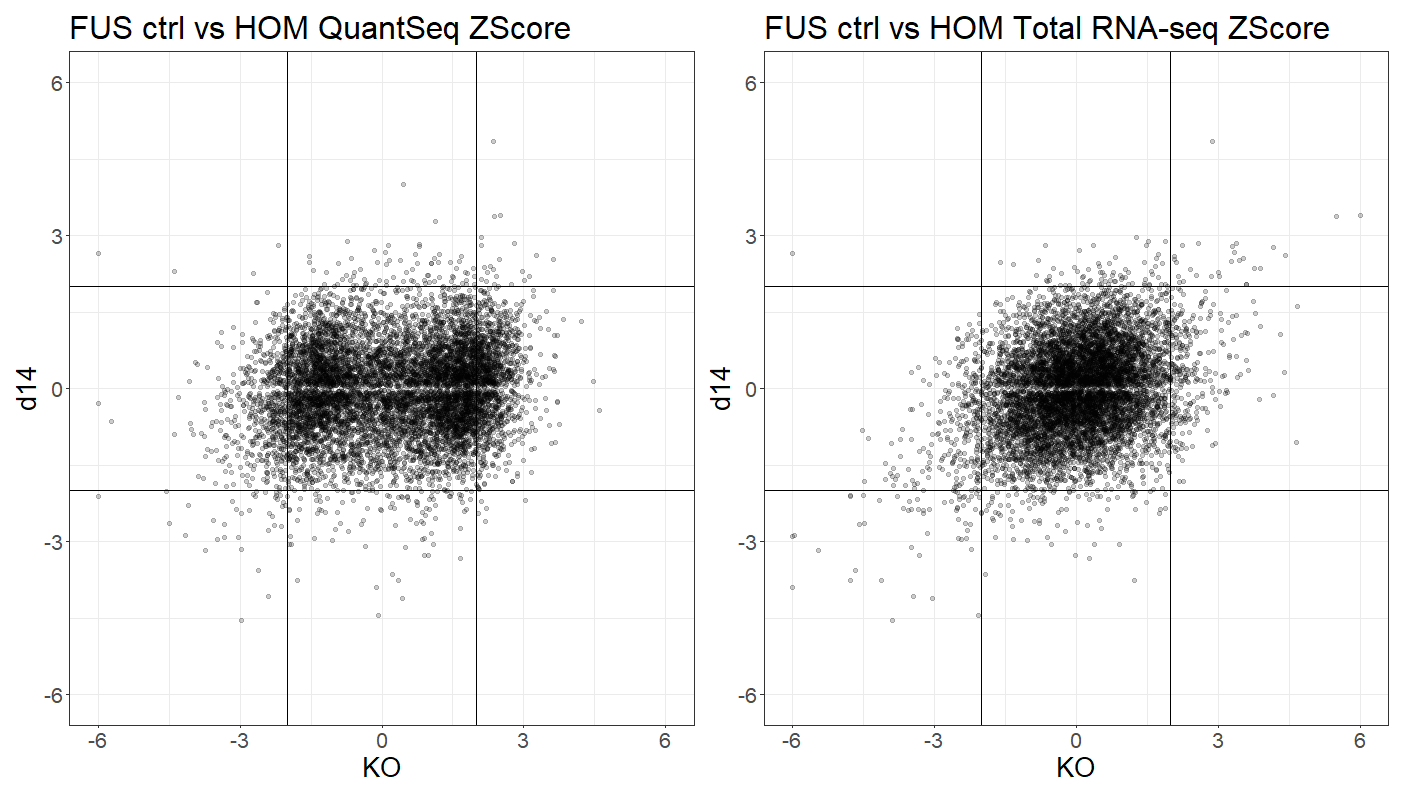


Supplementary Figure 4. Plots comparing Z-scores of d14 and KO datasets in QuantSeq and RNA-seq


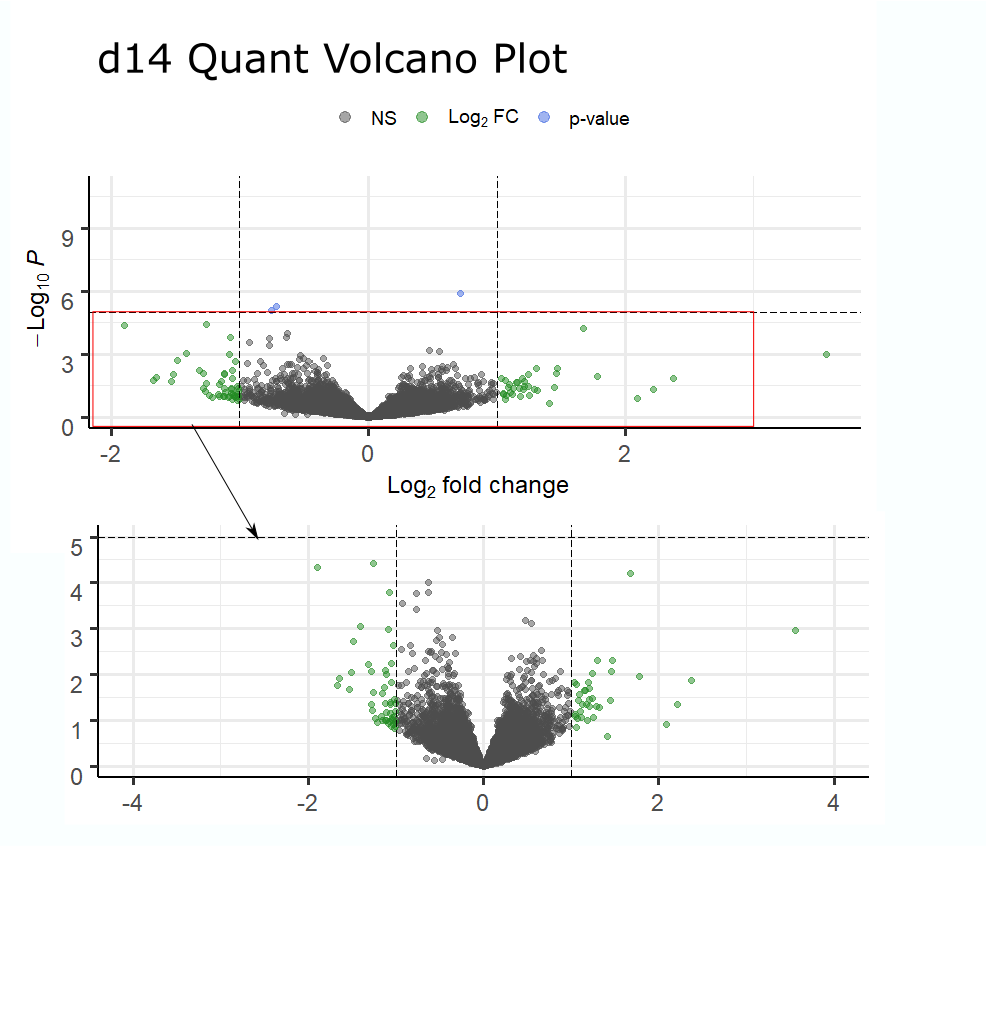


Supplementary Figure 5. Volcano plot showing distribution of genes in our d14 QuantSeq dataset. The cut-offs are set at a 2-fold change in expression and a p-value of 0.05, with green genes meeting the fold change threshold, blue meeting the p-value threshold, and red genes meeting both. The second part of the graph shows an expanded view of the area between -4 and 4 in the fold change and below 0.05 in the p-value.


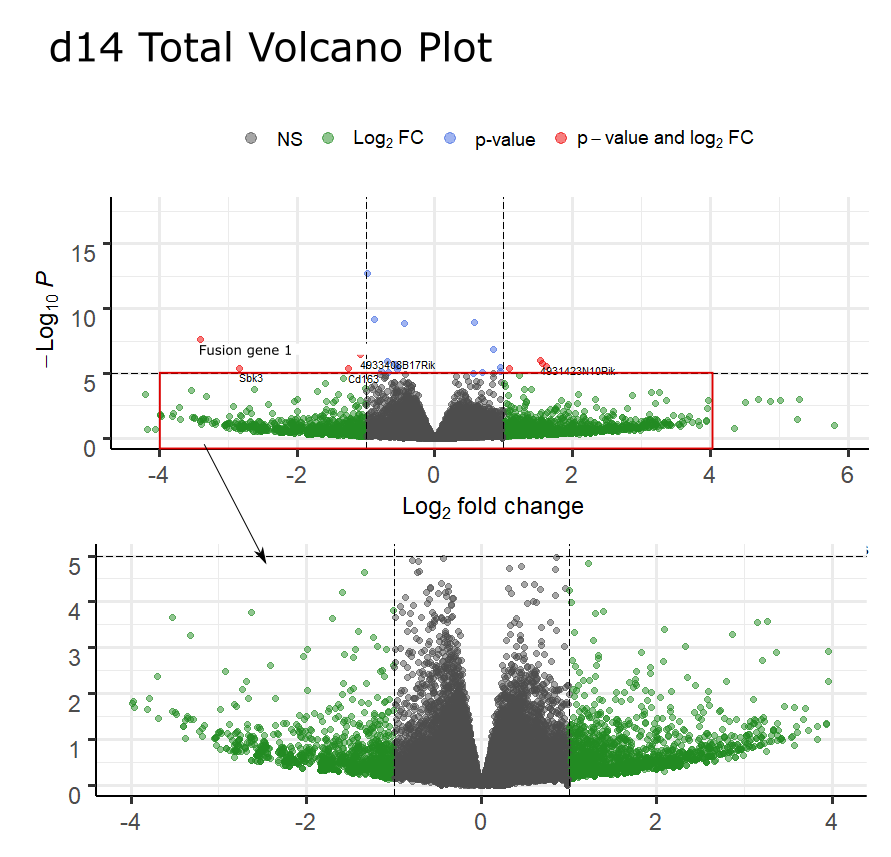


Supplementary Figure 6. Volcano plot showing distribution of genes in our d14 RNA-seq dataset. The cut-offs are set at a 2-fold change in expression and a p-value of 0.05, with green genes meeting the fold change threshold, blue meeting the p-value threshold, and red genes meeting both. The second part of the graph shows an expanded view of the area between -4 and 4 in the fold change and below 0.05 in the p-value. Fusion gene 1 is a combination of the genes 1700030C10Rik, Gm28503, and 1700030C10Rik


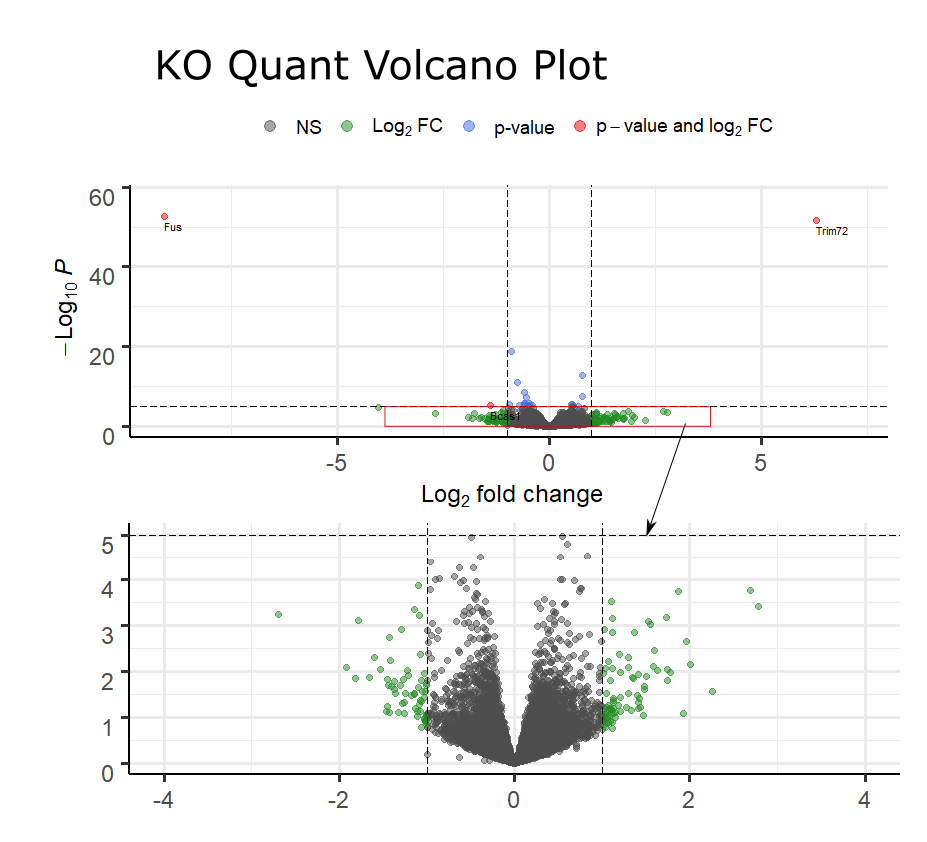


Supplementary Figure 7. Volcano plot showing distribution of genes in our KO QuantSeq dataset. The cut-offs are set at a 2-fold change in expression and a p-value of 0.05, with green genes meeting the fold change threshold, blue meeting the p-value threshold, and red genes meeting both. The second part of the graph shows an expanded view of the area between -4 and 4 in the fold change and below 0.05 in the p-value.


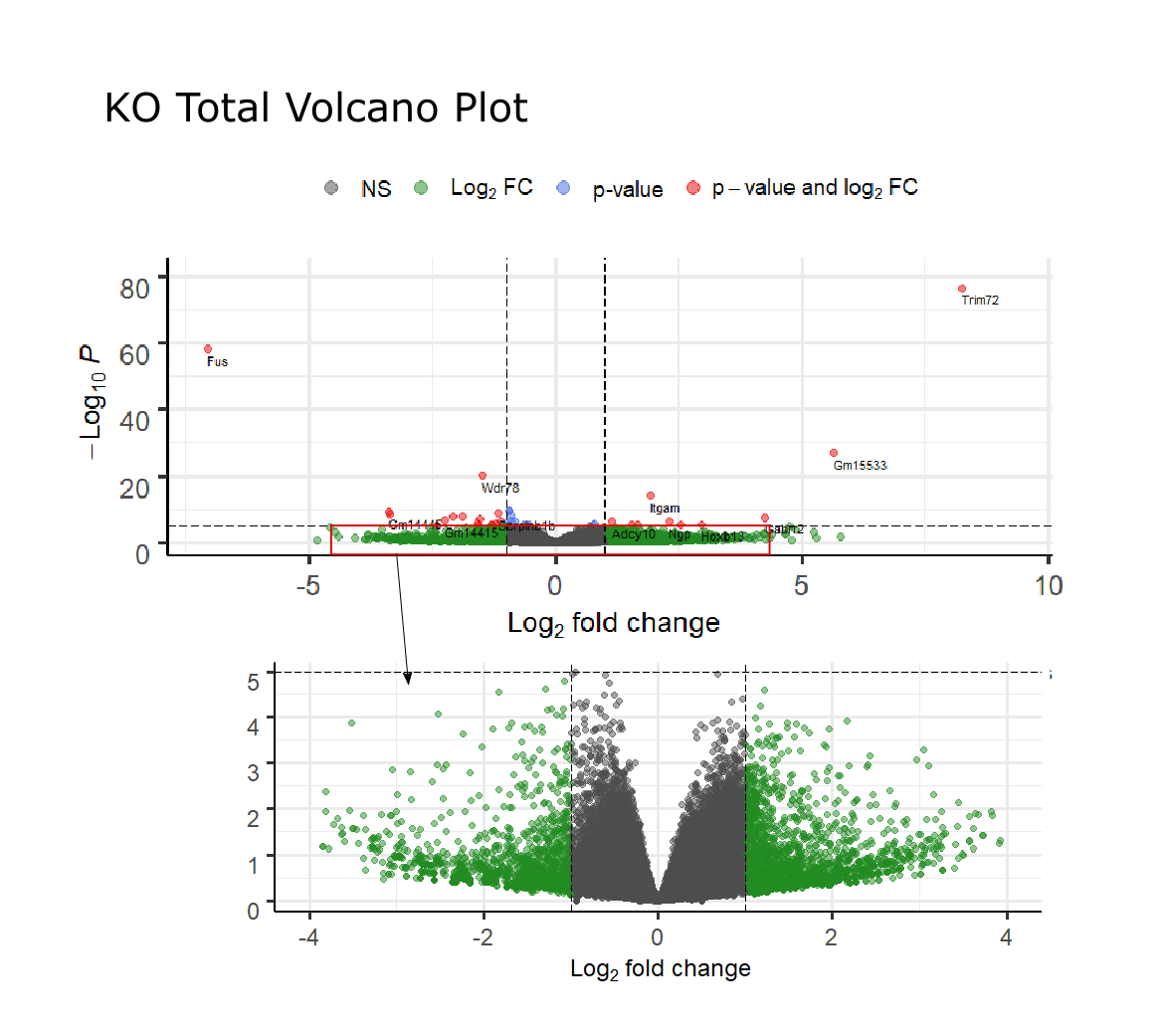


Supplementary Figure 8. Volcano plot showing distribution of genes in our KO RNA-seq dataset. The cut-offs are set at a 2-fold change in expression and a p-value of 0.05, with green genes meeting the fold change threshold, blue meeting the p-value threshold, and red genes meeting both. The second part of the graph shows an expanded view of the area between -4 and 4 in the fold change and below 0.05 in the p-value.


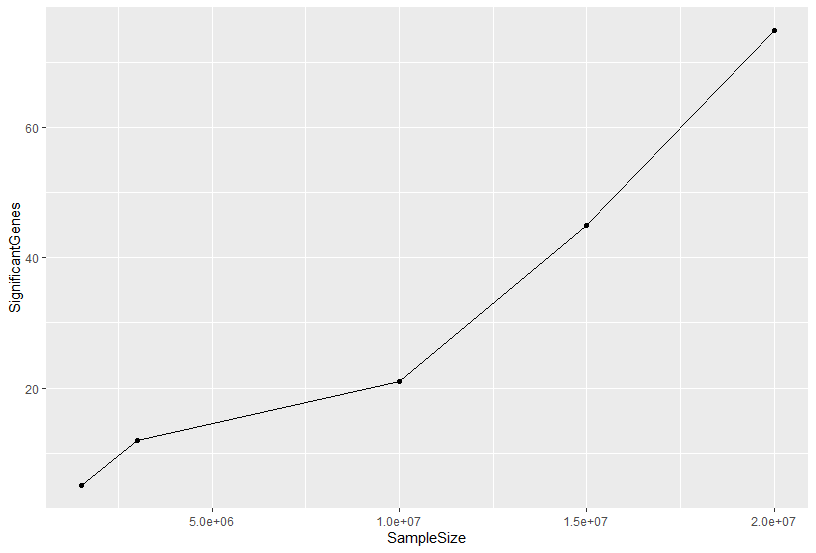
Supplementary Figure 9. Plot showing the number of genes with padj < 0.05 plotted against the number of reads that the KO RNA-seq dataset had been downsampled to


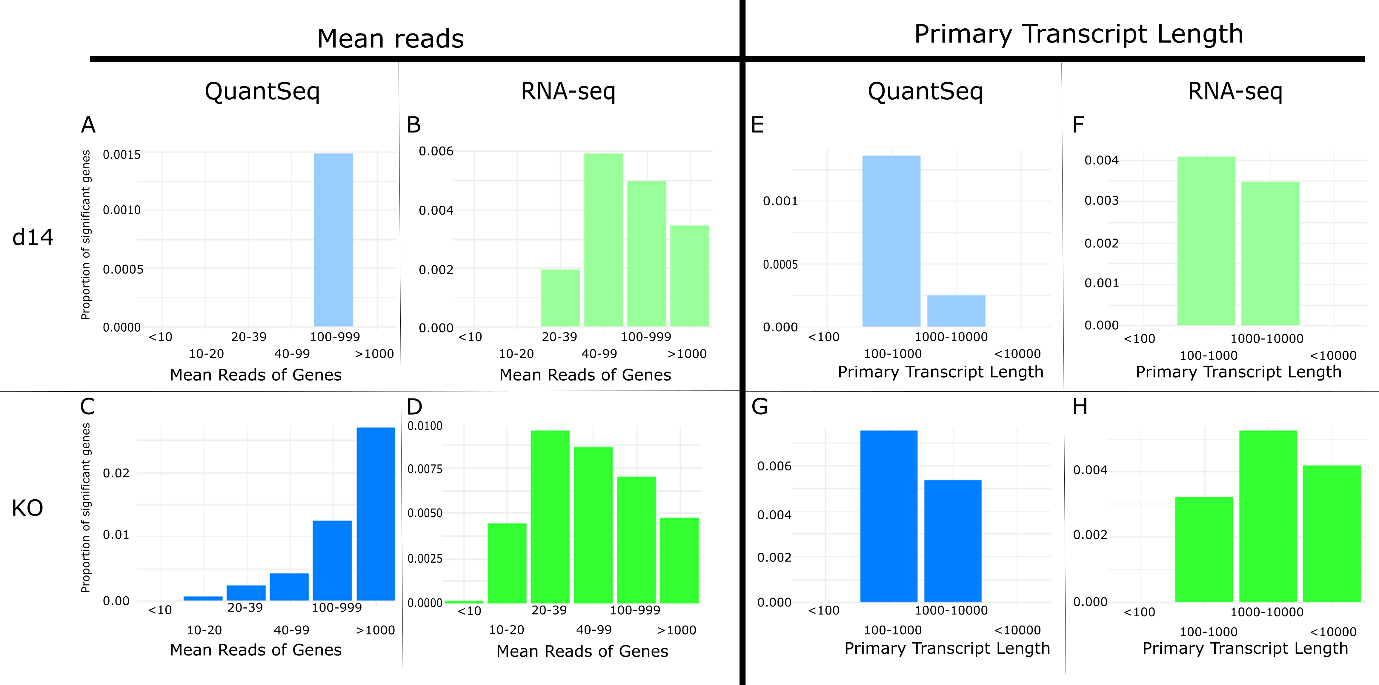
Supplementary Figure 10. (A-D) Bar plots showing the proportion of genes that are significantly differentially expressed at an adjusted level (padj < 0.05) separated by the mean number of reads in the gene using (A and C) QuantSeq, and (B and D) RNA-seq sequencing; (E-H) Bar plots showing the proportion of genes that are significantly differentially expressed (p-value < 0.05) separated by the length of the Appris Primary 1 transcript in the gene using the (E and G) QuantSeq, and (F and H) RNA-seq sequencing.


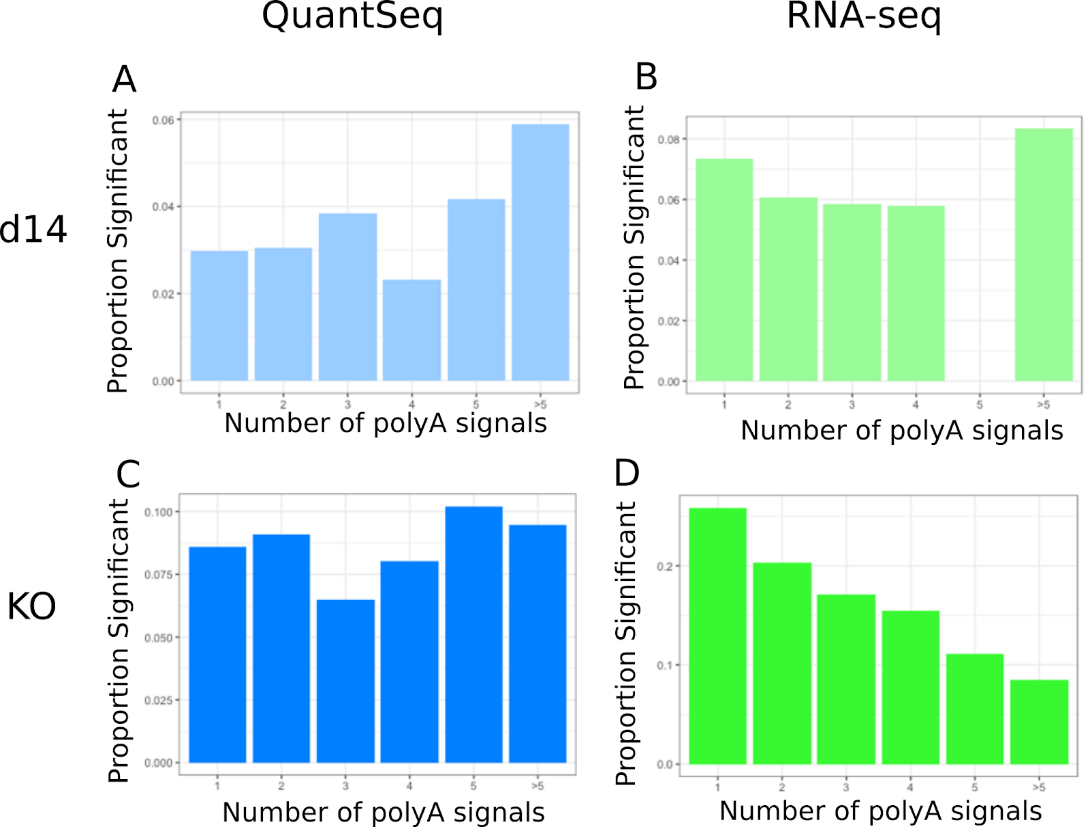


Supplementary Figure 11. Bar plots showing the proportion of genes that are significantly differentially expressed (p-value < 0.05) separated by the number of polyA signals each gene has found within the polyA atlas.

**
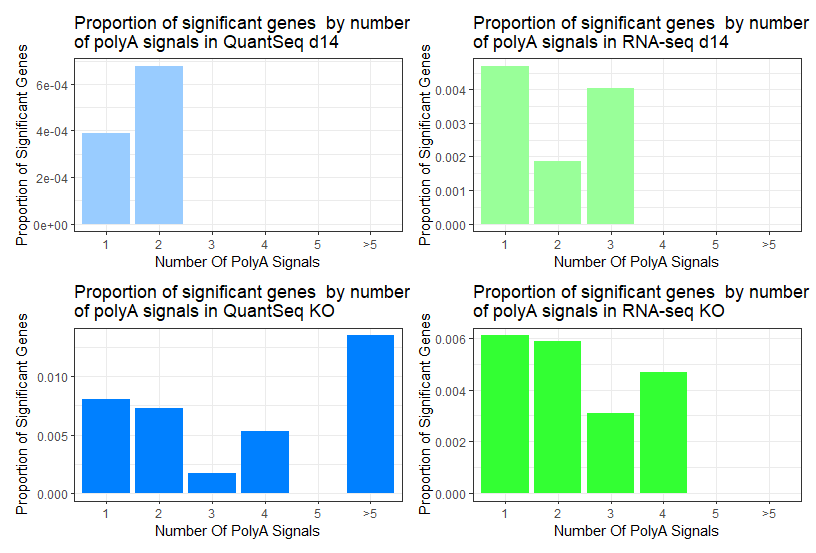
**

Supplementary Figure 12. Bar plots showing the proportion of genes that are significantly differentially expressed at an adjusted level (padj < 0.05) separated by the number of polyA signals each gene has found within the polyA atlas.


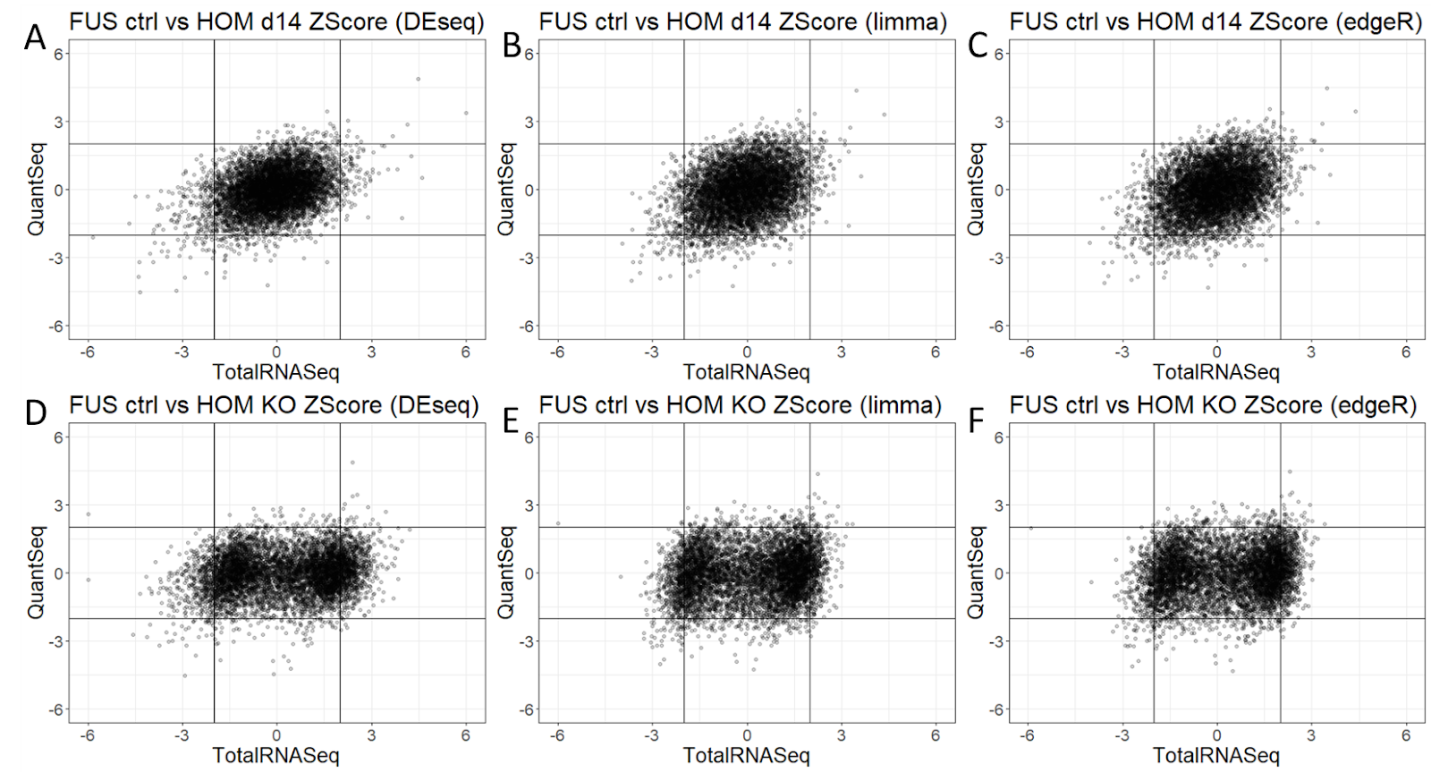


Supplementary Figure 13. Plots comparing Z-scores of RNA-seq and QuantSeq in d14 & KO experiments with RNA-seq and QuantSeq using the same DE method

 
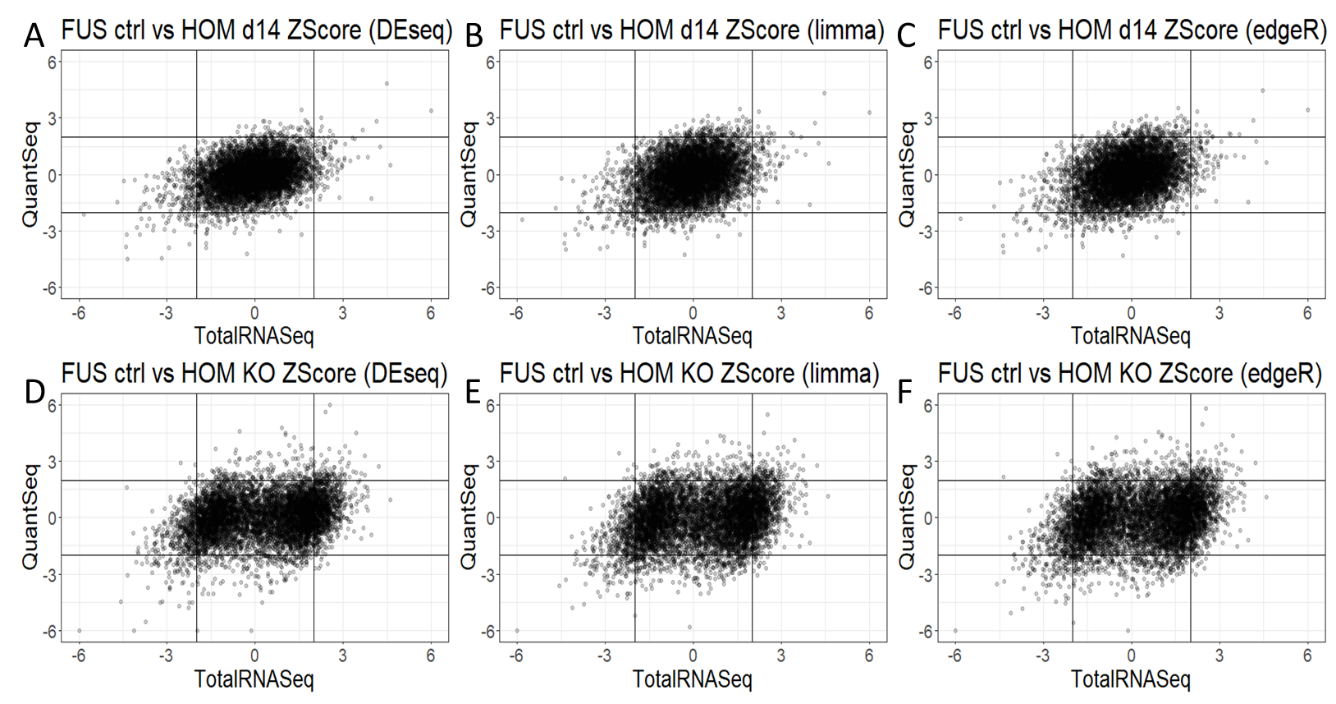


Supplementary Figure 14. Plots comparing Z-Scores of RNA-Seq and QuantSeq in d14 & KO experiments with RNA-seq and QuantSeq using the same DE method


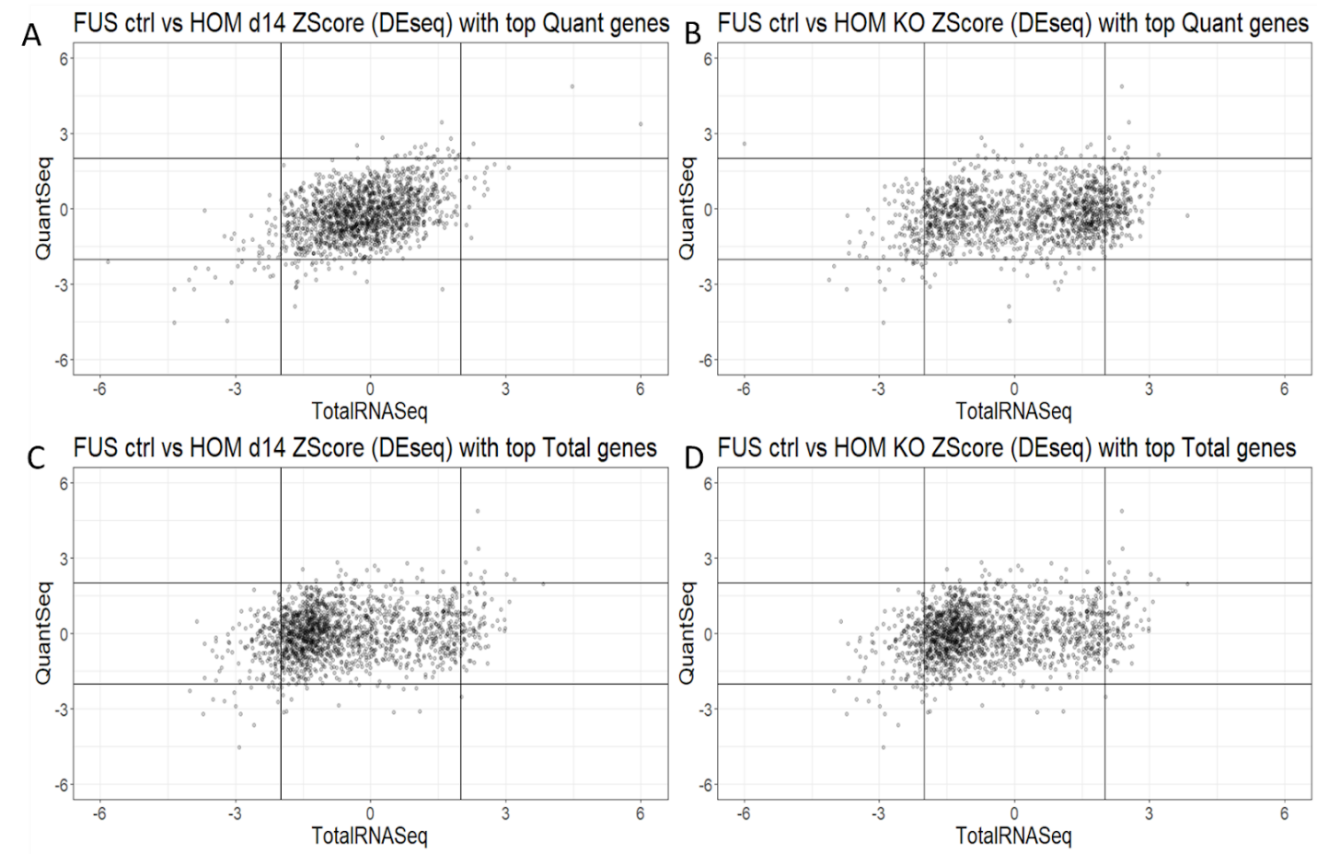


Supplementary Figure 15. Plots comparing Z-Scores of RNA-Seq and QuantSeq in d14 & KO experiments of the top 25% most expressed genes in QuantSeq and RNA-seq


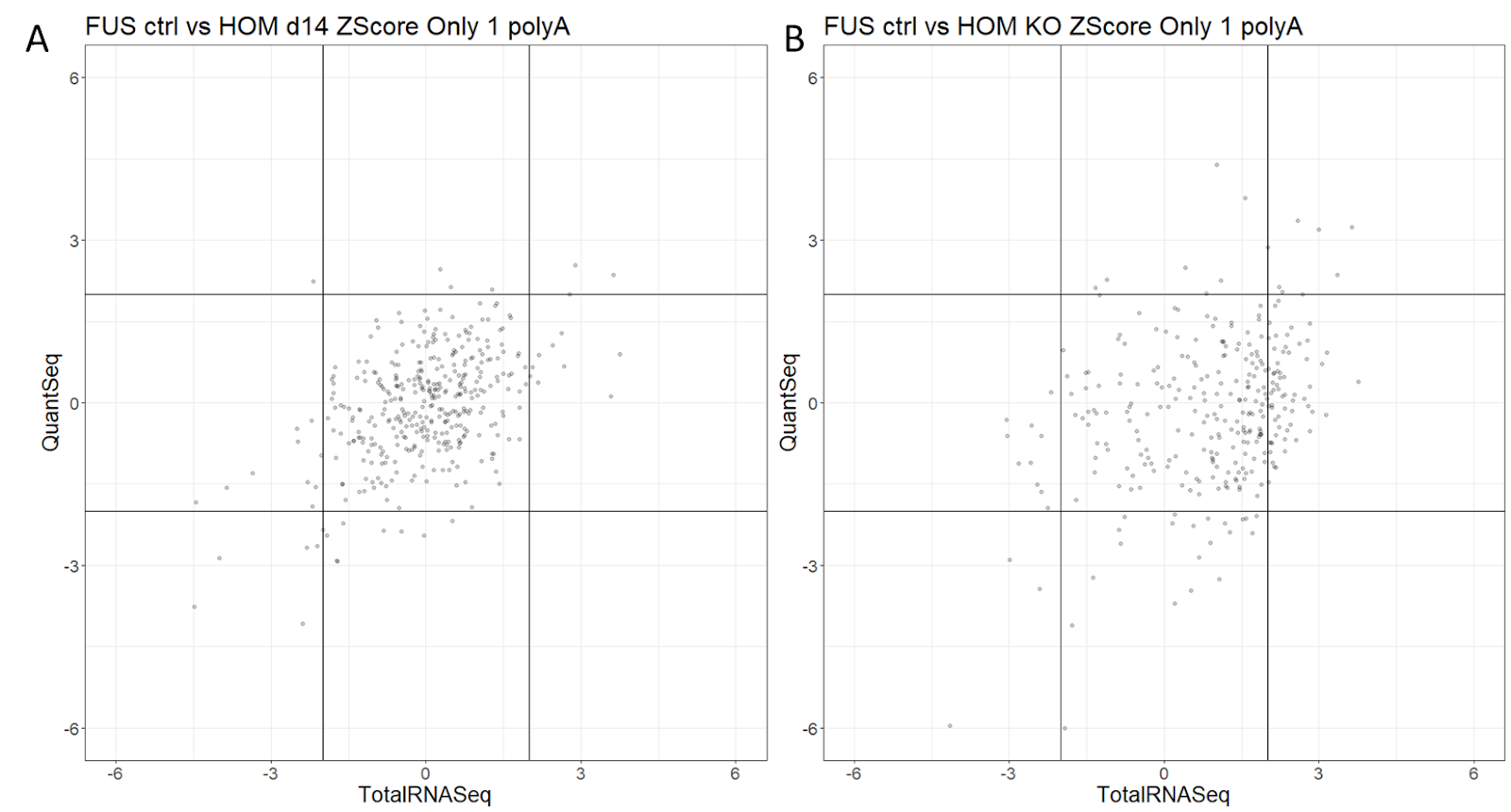


Supplementary Figure 16. Plots comparing Z-Scores of RNA-Seq and QuantSeq in d14 & KO experiments of genes which only have 1 polyA signal

Supplementary Table 1. Results of GO term analysis of all genes in d14 in both datasets

| Description | GO Term | Fold Enrichment Total | FDR Total | Fold Enrichment Quant | FDR Quant |
| --- | --- | --- | --- | --- | --- |
| sensory perception of chemical stimulus | GO:0007606 | -0.13 | 1.13E-10 | -NA | 0.00518 |
| sensory perception of smell | GO:0007608 | -0.12 | 3.36E-10 | -NA | 0.0115 |
| small molecule metabolic process | GO:0044281 | +1.72 | 0.0012 | +1.37 | 1 |
| sensory perception | GO:0007600 | -0.46 | 0.00142 | -0.62 | 1 |
| myofibril assembly | GO:0030239 | +5.36 | 0.00601 | +3.04 | 1 |
| regulation of biological quality | GO:0065008 | +1.34 | 0.01 | +1.69 | 0.00665 |
| lipid metabolic process | GO:0006629 | +1.74 | 0.0114 | +1.05 | 1 |
| cellular process | GO:0009987 | +1.11 | 0.0182 | +1.25 | 0.0013 |
| carboxylic acid metabolic process | GO:0019752 | +1.85 | 0.0248 | +1.1 | 1 |
| response to chemical | GO:0042221 | +1.34 | 0.0254 | +1.5 | 0.235 |
| developmental process | GO:0032502 | +1.25 | 0.0256 | +1.62 | 0.00141 |
| oxidation-reduction process | GO:0055114 | +1.82 | 0.0258 | -0.95 | 1 |
| cellular lipid metabolic process | GO:0044255 | +1.82 | 0.0258 | +1.16 | 1 |
| metabolic process | GO:0008152 | +1.2 | 0.0305 | +1.31 | 0.195 |
| organic acid metabolic process | GO:0006082 | +1.78 | 0.0312 | +1.35 | 1 |
| cellular component assembly involved in morphogenesis | GO:0010927 | +3.63 | 0.0331 | +1.8 | 1 |
| transmembrane transport | GO:0055085 | +1.72 | 0.0485 | +1.47 | 1 |
| anatomical structure development | GO:0048856 | +1.21 | 0.202 | +1.55 | 0.012 |
| multicellular organism development | GO:0007275 | +1.23 | 0.204 | +1.58 | 0.0121 |
| system development | GO:0048731 | +1.24 | 0.273 | +1.63 | 0.0119 |
| Unclassified | UNCLASSIFIED | -0.66 | 0.333 | -0.24 | 0.0326 |
| cell differentiation | GO:0030154 | +1.25 | 0.336 | +1.63 | 0.0323 |
| biological_process | GO:0008150 | +1.03 | 0.339 | +1.07 | 0.0315 |
| cellular chemical homeostasis | GO:0055082 | +1.61 | 0.346 | +2.77 | 0.0207 |
| cellular homeostasis | GO:0019725 | +1.56 | 0.381 | +2.49 | 0.0382 |
| negative regulation of biological process | GO:0048519 | +1.2 | 0.382 | +1.67 | 0.00132 |
| cellular developmental process | GO:0048869 | +1.24 | 0.387 | +1.65 | 0.0219 |
| cellular cation homeostasis | GO:0030003 | +1.65 | 0.442 | +2.76 | 0.044 |
| multicellular organismal process | GO:0032501 | +1.12 | 0.881 | +1.38 | 0.0388 |
| negative regulation of cellular process | GO:0048523 | +1.17 | 0.886 | +1.63 | 0.00614 |
| cell cycle G1/S phase transition | GO:0044843 | -0.97 | 1 | +10.31 | 0.00587 |
| regulation of secretion by cell | GO:1903530 | +1.37 | 1 | +2.89 | 0.0062 |
| G1/S transition of mitotic cell cycle | GO:0000082 | -0.67 | 1 | +10.6 | 0.00725 |
| regulation of secretion | GO:0051046 | +1.3 | 1 | +2.63 | 0.011 |
| regulation of cell population proliferation | GO:0042127 | +1.14 | 1 | +2.13 | 0.0122 |
| regulation of exocytosis | GO:0017157 | +1.13 | 1 | +4.49 | 0.0205 |
| biological adhesion | GO:0022610 | +1.29 | 1 | +2.67 | 0.0206 |
| secretion | GO:0046903 | +1.47 | 1 | +3.19 | 0.0213 |
| cell adhesion | GO:0007155 | +1.27 | 1 | +2.7 | 0.0213 |
| positive regulation of cell cycle process | GO:0090068 | +1.29 | 1 | +4.4 | 0.0214 |
| positive regulation of cellular process | GO:0048522 | +1.07 | 1 | +1.49 | 0.0282 |
| positive regulation of cell cycle | GO:0045787 | +1.05 | 1 | +3.65 | 0.0313 |
| response to metal ion | GO:0010038 | +1.74 | 1 | +4.15 | 0.0321 |
| regulation of regulated secretory pathway | GO:1903305 | -0.99 | 1 | +4.93 | 0.0325 |
| response to abiotic stimulus | GO:0009628 | +1.36 | 1 | +2.45 | 0.0489 |

Supplementary Table 2. Results of GO term analysis of all genes in KO in both datasets

| Description | GO Term | Fold Enrichment Total | FDR Total | Fold Enrichment Quant | FDR Quant |
| --- | --- | --- | --- | --- | --- |
| sensory perception of smell | GO:0007608 | -0.06 | 1.24E-44 | -NA | 4.04E-10 |
| sensory perception of chemical stimulus | GO:0007606 | -0.09 | 1.22E-43 | -0.03 | 3.47E-10 |
| metabolic process | GO:0008152 | +1.39 | 4.85E-36 | +1.42 | 9.88E-09 |
| cellular metabolic process | GO:0044237 | +1.4 | 3.21E-32 | +1.46 | 2.23E-08 |
| organic substance metabolic process | GO:0071704 | +1.38 | 3.63E-31 | +1.4 | 6.44E-07 |
| primary metabolic process | GO:0044238 | +1.39 | 3.84E-29 | +1.4 | 2.32E-06 |
| nitrogen compound metabolic process | GO:0006807 | +1.41 | 3.78E-28 | +1.42 | 6.10E-06 |
| sensory perception | GO:0007600 | -0.34 | 2.11E-24 | -0.41 | 0.00142 |
| cellular process | GO:0009987 | +1.15 | 2.64E-21 | +1.2 | 1.39E-07 |
| macromolecule metabolic process | GO:0043170 | +1.38 | 5.32E-21 | +1.49 | 7.06E-07 |
| cellular nitrogen compound metabolic process | GO:0034641 | +1.54 | 1.16E-18 | +1.51 | 0.00152 |
| G protein-coupled receptor signaling pathway | GO:0007186 | -0.45 | 2.78E-18 | -0.5 | 0.0101 |
| biosynthetic process | GO:0009058 | +1.61 | 2.19E-17 | +1.34 | 0.256 |
| organonitrogen compound metabolic process | GO:1901564 | +1.39 | 6.18E-17 | +1.39 | 0.00121 |
| cellular biosynthetic process | GO:0044249 | +1.61 | 4.94E-16 | +1.39 | 0.177 |
| organic substance biosynthetic process | GO:1901576 | +1.59 | 6.84E-16 | +1.34 | 0.305 |
| cellular component organization or biogenesis | GO:0071840 | +1.32 | 8.05E-16 | +1.43 | 1.17E-05 |
| cellular macromolecule metabolic process | GO:0044260 | +1.38 | 2.28E-14 | +1.52 | 1.03E-05 |
| nervous system process | GO:0050877 | -0.53 | 3.05E-14 | -0.84 | 1 |
| cellular component organization | GO:0016043 | +1.3 | 3.95E-13 | +1.42 | 3.25E-05 |
| heterocycle metabolic process | GO:0046483 | +1.47 | 1.95E-11 | +1.41 | 0.0739 |
| biological_process | GO:0008150 | +1.04 | 2.57E-11 | +1.05 | 0.00944 |
| Unclassified | UNCLASSIFIED | -0.55 | 2.69E-11 | -0.51 | 0.00936 |
| protein metabolic process | GO:0019538 | +1.37 | 2.97E-11 | +1.45 | 0.00166 |
| cellular nitrogen compound biosynthetic process | GO:0044271 | +1.72 | 3.24E-11 | +1.48 | 0.34 |
| nucleobase-containing compound metabolic process | GO:0006139 | +1.47 | 6.38E-11 | +1.48 | 0.0235 |
| cellular aromatic compound metabolic process | GO:0006725 | +1.45 | 6.50E-11 | +1.39 | 0.0824 |
| cellular component biogenesis | GO:0044085 | +1.46 | 8.57E-11 | +1.6 | 0.000813 |
| localization | GO:0051179 | +1.28 | 1.07E-10 | +1.45 | 1.33E-05 |
| organonitrogen compound biosynthetic process | GO:1901566 | +1.71 | 1.85E-10 | +1.63 | 0.077 |
| organelle organization | GO:0006996 | +1.38 | 1.90E-10 | +1.61 | 1.36E-05 |
| cellular protein metabolic process | GO:0044267 | +1.39 | 2.76E-10 | +1.6 | 4.53E-05 |
| organic cyclic compound metabolic process | GO:1901360 | +1.41 | 2.93E-10 | +1.36 | 0.0939 |
| gene expression | GO:0010467 | +1.52 | 2.46E-09 | +1.86 | 3.58E-05 |
| cellular localization | GO:0051641 | +1.45 | 5.39E-09 | +1.42 | 0.103 |
| amide biosynthetic process | GO:0043604 | +2.11 | 8.09E-09 | +2.05 | 0.0893 |
| negative regulation of biological process | GO:0048519 | +1.24 | 1.48E-08 | +1.37 | 0.000319 |
| regulation of protein metabolic process | GO:0051246 | +1.36 | 1.90E-08 | +1.51 | 0.00199 |
| establishment of localization | GO:0051234 | +1.3 | 2.32E-08 | +1.52 | 2.93E-05 |
| nucleic acid metabolic process | GO:0090304 | +1.47 | 2.32E-08 | +1.55 | 0.0186 |
| cellular amide metabolic process | GO:0043603 | +1.83 | 2.34E-08 | +1.78 | 0.101 |
| transport | GO:0006810 | +1.31 | 2.40E-08 | +1.51 | 7.08E-05 |
| regulation of biological quality | GO:0065008 | +1.29 | 2.74E-08 | +1.58 | 7.05E-07 |
| regulation of metabolic process | GO:0019222 | +1.21 | 5.58E-08 | +1.32 | 0.00117 |
| cellular component assembly | GO:0022607 | +1.42 | 9.91E-08 | +1.59 | 0.00387 |
| RNA metabolic process | GO:0016070 | +1.54 | 2.39E-07 | +1.71 | 0.0115 |
| system process | GO:0003008 | -0.68 | 2.45E-07 | -0.86 | 1 |
| humoral immune response mediated by circulating immunoglobulin | GO:0002455 | -0.07 | 3.82E-07 | -NA | 0.199 |
| regulation of localization | GO:0032879 | +1.32 | 4.31E-07 | +1.68 | 2.06E-06 |
| macromolecule localization | GO:0033036 | +1.37 | 4.76E-07 | +1.51 | 0.00975 |
| complement activation, classical pathway | GO:0006958 | -0.07 | 4.97E-07 | -NA | 0.193 |
| regulation of cellular metabolic process | GO:0031323 | +1.21 | 5.72E-07 | +1.34 | 0.000916 |
| regulation of cellular protein metabolic process | GO:0032268 | +1.34 | 8.21E-07 | +1.54 | 0.00191 |
| translation | GO:0006412 | +2.15 | 1.01E-06 | +2.7 | 0.00541 |
| humoral immune response | GO:0006959 | -0.27 | 1.02E-06 | -0.27 | 0.266 |
| complement activation | GO:0006956 | -0.1 | 1.32E-06 | -NA | 0.146 |
| peptide biosynthetic process | GO:0043043 | +2.09 | 1.70E-06 | +2.54 | 0.0108 |
| small molecule metabolic process | GO:0044281 | +1.47 | 1.72E-06 | +1.25 | 0.915 |
| phagocytosis, recognition | GO:0006910 | -0.08 | 2.05E-06 | -NA | 0.25 |
| small molecule biosynthetic process | GO:0044283 | +1.89 | 2.57E-06 | +1.28 | 1 |
| regulation of nitrogen compound metabolic process | GO:0051171 | +1.21 | 2.63E-06 | +1.32 | 0.00392 |
| regulation of primary metabolic process | GO:0080090 | +1.2 | 3.13E-06 | +1.36 | 0.000405 |
| negative regulation of cellular process | GO:0048523 | +1.22 | 3.18E-06 | +1.41 | 0.000214 |
| regulation of macromolecule metabolic process | GO:0060255 | +1.2 | 3.42E-06 | +1.31 | 0.00421 |
| B cell receptor signaling pathway | GO:0050853 | -0.11 | 3.66E-06 | -NA | 0.193 |
| organic substance transport | GO:0071702 | +1.39 | 5.35E-06 | +1.67 | 0.000857 |
| macromolecule biosynthetic process | GO:0009059 | +1.49 | 6.34E-06 | +1.51 | 0.177 |
| peptide metabolic process | GO:0006518 | +1.86 | 7.06E-06 | +2.16 | 0.0273 |
| immunoglobulin production | GO:0002377 | -0.18 | 7.55E-06 | -0.14 | 0.328 |
| phagocytosis, engulfment | GO:0006911 | -0.14 | 7.63E-06 | -0.16 | 0.611 |
| regulation of cellular component organization | GO:0051128 | +1.31 | 9.60E-06 | +1.85 | 2.80E-08 |
| positive regulation of biological process | GO:0048518 | +1.18 | 1.08E-05 | +1.36 | 4.61E-05 |
| ncRNA metabolic process | GO:0034660 | +1.88 | 1.10E-05 | +1.63 | 0.731 |
| positive regulation of cellular process | GO:0048522 | +1.19 | 1.14E-05 | +1.4 | 2.90E-05 |
| RNA processing | GO:0006396 | +1.63 | 1.14E-05 | +2.04 | 0.00352 |
| production of molecular mediator of immune response | GO:0002440 | -0.22 | 1.14E-05 | -0.13 | 0.274 |
| nitrogen compound transport | GO:0071705 | +1.42 | 1.19E-05 | +1.78 | 0.000441 |
| cellular macromolecule biosynthetic process | GO:0034645 | +1.48 | 1.50E-05 | +1.56 | 0.112 |
| protein localization | GO:0008104 | +1.36 | 1.72E-05 | +1.51 | 0.0248 |
| intracellular transport | GO:0046907 | +1.48 | 1.87E-05 | +1.58 | 0.0879 |
| defense response to bacterium | GO:0042742 | -0.38 | 2.74E-05 | -0.07 | 0.00654 |
| protein-containing complex subunit organization | GO:0043933 | +1.45 | 3.19E-05 | +1.86 | 0.000696 |
| positive regulation of protein metabolic process | GO:0051247 | +1.38 | 5.29E-05 | +1.43 | 0.176 |
| amide transport | GO:0042886 | +1.43 | 6.58E-05 | +1.62 | 0.0346 |
| protein-containing complex assembly | GO:0065003 | +1.47 | 6.70E-05 | +2.03 | 8.67E-05 |
| macromolecule modification | GO:0043412 | +1.3 | 7.07E-05 | +1.41 | 0.0519 |
| plasma membrane invagination | GO:0099024 | -0.2 | 7.35E-05 | -0.15 | 0.488 |
| catabolic process | GO:0009056 | +1.36 | 8.01E-05 | +1.23 | 0.858 |
| peptide transport | GO:0015833 | +1.43 | 9.43E-05 | +1.62 | 0.0404 |
| multicellular organism development | GO:0007275 | +1.19 | 0.000103 | +1.49 | 1.50E-06 |
| protein transport | GO:0015031 | +1.43 | 0.000131 | +1.66 | 0.0271 |
| rRNA processing | GO:0006364 | +2.21 | 0.000151 | +1.45 | 1 |
| lipid biosynthetic process | GO:0008610 | +1.78 | 0.000151 | -0.78 | 1 |
| phosphorus metabolic process | GO:0006793 | +1.36 | 0.000151 | +1.18 | 1 |
| phosphate-containing compound metabolic process | GO:0006796 | +1.36 | 0.000154 | +1.17 | 1 |
| immunoglobulin mediated immune response | GO:0016064 | -0.26 | 0.000162 | -NA | 0.0661 |
| membrane invagination | GO:0010324 | -0.22 | 0.000166 | -0.15 | 0.5 |
| positive regulation of cellular protein metabolic process | GO:0032270 | +1.37 | 0.000167 | +1.47 | 0.146 |
| ncRNA processing | GO:0034470 | +1.88 | 0.000186 | +1.68 | 0.7 |
| establishment of protein localization | GO:0045184 | +1.4 | 0.000203 | +1.69 | 0.0102 |
| rRNA metabolic process | GO:0016072 | +2.15 | 0.000232 | +1.39 | 1 |
| cellular catabolic process | GO:0044248 | +1.37 | 0.000261 | +1.31 | 0.668 |
| cellular protein localization | GO:0034613 | +1.38 | 0.000269 | +1.36 | 0.506 |
| cellular macromolecule localization | GO:0070727 | +1.38 | 0.00028 | +1.37 | 0.455 |
| cellular lipid metabolic process | GO:0044255 | +1.52 | 0.000311 | +1.3 | 1 |
| B cell mediated immunity | GO:0019724 | -0.28 | 0.000314 | -NA | 0.0668 |
| positive regulation of metabolic process | GO:0009893 | +1.22 | 0.000328 | +1.27 | 0.213 |
| extracellular structure organization | GO:0043062 | +2.02 | 0.000347 | +1.63 | 0.914 |
| intracellular signal transduction | GO:0035556 | +1.38 | 0.000384 | +1.3 | 0.768 |
| developmental process | GO:0032502 | +1.16 | 0.000425 | +1.43 | 3.68E-06 |
| positive regulation of B cell activation | GO:0050871 | -0.28 | 0.000426 | -0.53 | 1 |
| regulation of transport | GO:0051049 | +1.31 | 0.000427 | +1.86 | 2.53E-06 |
| regulation of cell communication | GO:0010646 | +1.23 | 0.000428 | +1.6 | 7.35E-06 |
| regulation of signaling | GO:0023051 | +1.23 | 0.000431 | +1.6 | 9.69E-06 |
| oxidation-reduction process | GO:0055114 | +1.52 | 0.000481 | +1.46 | 0.622 |
| regulation of molecular function | GO:0065009 | +1.26 | 0.000484 | +1.43 | 0.0266 |
| negative regulation of metabolic process | GO:0009892 | +1.25 | 0.000504 | +1.47 | 0.0057 |
| anatomical structure development | GO:0048856 | +1.17 | 0.000525 | +1.43 | 9.27E-06 |
| regulation of organelle organization | GO:0033043 | +1.39 | 0.000546 | +1.73 | 0.00828 |
| protein modification process | GO:0036211 | +1.28 | 0.000549 | +1.41 | 0.0769 |
| response to pheromone | GO:0019236 | -NA | 0.00055 | -NA | 0.941 |
| cellular protein modification process | GO:0006464 | +1.28 | 0.000553 | +1.41 | 0.0772 |
| regulation of catabolic process | GO:0009894 | +1.49 | 0.000584 | +1.61 | 0.212 |
| extracellular matrix organization | GO:0030198 | +1.99 | 0.000635 | +1.63 | 0.907 |
| positive regulation of cellular metabolic process | GO:0031325 | +1.22 | 0.00071 | +1.22 | 0.506 |
| positive regulation of cellular component organization | GO:0051130 | +1.39 | 0.000782 | +2.1 | 4.29E-06 |
| transmembrane transport | GO:0055085 | +1.46 | 0.000931 | +1.82 | 0.0194 |
| system development | GO:0048731 | +1.19 | 0.00102 | +1.54 | 1.54E-06 |
| regulation of cellular amide metabolic process | GO:0034248 | +1.74 | 0.00103 | +2.23 | 0.0433 |
| organic substance catabolic process | GO:1901575 | +1.35 | 0.00106 | +1.29 | 0.802 |
| carboxylic acid metabolic process | GO:0019752 | +1.5 | 0.00108 | +1.36 | 0.853 |
| posttranscriptional regulation of gene expression | GO:0010608 | +1.69 | 0.00109 | +2.09 | 0.0678 |
| regulation of catalytic activity | GO:0050790 | +1.3 | 0.00127 | +1.38 | 0.245 |
| cellular protein-containing complex assembly | GO:0034622 | +1.54 | 0.00134 | +1.76 | 0.125 |
| oxoacid metabolic process | GO:0043436 | +1.48 | 0.00138 | +1.33 | 0.973 |
| regulation of translation | GO:0006417 | +1.78 | 0.00142 | +2.25 | 0.059 |
| establishment of localization in cell | GO:0051649 | +1.77 | 0.00158 | +1.26 | 1 |
| sphingolipid metabolic process | GO:0006665 | +2.38 | 0.00179 | +1.47 | 1 |
| negative regulation of macromolecule metabolic process | GO:0010605 | +1.25 | 0.00182 | +1.5 | 0.0057 |
| organic acid metabolic process | GO:0006082 | +1.46 | 0.00221 | +1.29 | 1 |
| lipid metabolic process | GO:0006629 | +1.4 | 0.00231 | +1.25 | 1 |
| ribosome biogenesis | GO:0042254 | +1.82 | 0.0026 | +1.38 | 1 |
| organic cyclic compound biosynthetic process | GO:1901362 | +1.45 | 0.00262 | -0.97 | 1 |
| response to organonitrogen compound | GO:0010243 | +1.48 | 0.00264 | +1.68 | 0.184 |
| positive regulation of macromolecule metabolic process | GO:0010604 | +1.21 | 0.00265 | +1.27 | 0.246 |
| nervous system development | GO:0007399 | +1.26 | 0.00268 | +2.01 | 5.29E-10 |
| ribonucleoprotein complex biogenesis | GO:0022613 | +1.69 | 0.0028 | +1.46 | 1 |
| negative regulation of nitrogen compound metabolic process | GO:0051172 | +1.25 | 0.00281 | +1.54 | 0.00409 |
| carbohydrate derivative metabolic process | GO:1901135 | +1.46 | 0.00293 | +1.03 | 1 |
| regulation of B cell activation | GO:0050864 | -0.39 | 0.00305 | -0.57 | 1 |
| response to nitrogen compound | GO:1901698 | +1.44 | 0.00322 | +1.55 | 0.324 |
| organic acid biosynthetic process | GO:0016053 | +1.93 | 0.00323 | +1.75 | 0.79 |
| phosphorylation | GO:0016310 | +1.4 | 0.00416 | +1.29 | 0.992 |
| regulation of response to stimulus | GO:0048583 | +1.18 | 0.00418 | +1.25 | 0.193 |
| carboxylic acid biosynthetic process | GO:0046394 | +1.91 | 0.00459 | +1.76 | 0.794 |
| regulation of signal transduction | GO:0009966 | +1.22 | 0.0046 | +1.43 | 0.0164 |
| ion transmembrane transport | GO:0034220 | +1.52 | 0.00501 | +1.87 | 0.0881 |
| heterocycle biosynthetic process | GO:0018130 | +1.47 | 0.00545 | -1 | 1 |
| regulation of cell migration | GO:0030334 | +1.41 | 0.00547 | +1.68 | 0.088 |
| regulation of supramolecular fiber organization | GO:1902903 | +1.67 | 0.00553 | +2.23 | 0.0526 |
| protein localization to organelle | GO:0033365 | +1.51 | 0.00559 | +1.32 | 1 |
| regulation of cellular component biogenesis | GO:0044087 | +1.39 | 0.00578 | +1.84 | 0.00912 |
| organonitrogen compound catabolic process | GO:1901565 | +1.41 | 0.00589 | +1.39 | 0.758 |
| regulation of cell death | GO:0010941 | +1.29 | 0.0059 | +1.36 | 0.372 |
| regulation of cellular component movement | GO:0051270 | +1.37 | 0.00598 | +1.66 | 0.0575 |
| positive regulation of cell migration | GO:0030335 | +1.54 | 0.00601 | +2 | 0.0373 |
| negative regulation of cellular metabolic process | GO:0031324 | +1.23 | 0.00616 | +1.52 | 0.00425 |
| antigen receptor-mediated signaling pathway | GO:0050851 | -0.4 | 0.00621 | -0.12 | 0.166 |
| membrane lipid metabolic process | GO:0006643 | +2.06 | 0.00628 | +1.32 | 1 |
| regulation of anatomical structure morphogenesis | GO:0022603 | +1.37 | 0.00634 | +2 | 0.000258 |
| positive regulation of cell motility | GO:2000147 | +1.52 | 0.00635 | +1.92 | 0.0603 |
| regulation of cell motility | GO:2000145 | +1.39 | 0.00638 | +1.59 | 0.166 |
| regulation of actin cytoskeleton organization | GO:0032956 | +1.67 | 0.00779 | +2.18 | 0.0739 |
| regulation of cellular catabolic process | GO:0031329 | +1.46 | 0.00789 | +1.54 | 0.522 |
| regulation of cytoskeleton organization | GO:0051493 | +1.52 | 0.0079 | +2.49 | 0.000179 |
| chemical homeostasis | GO:0048878 | +1.35 | 0.00895 | +1.66 | 0.0474 |
| negative regulation of response to stimulus | GO:0048585 | +1.29 | 0.00926 | +1.42 | 0.25 |
| regulation of protein modification process | GO:0031399 | +1.27 | 0.00929 | +1.55 | 0.0203 |
| homeostatic process | GO:0042592 | +1.28 | 0.00982 | +1.44 | 0.17 |
| intracellular protein transport | GO:0006886 | +1.43 | 0.01 | +1.54 | 0.437 |
| cell adhesion | GO:0007155 | +1.42 | 0.0102 | +1.81 | 0.0502 |
| positive regulation of catalytic activity | GO:0043085 | +1.35 | 0.0108 | +1.43 | 0.502 |
| biological adhesion | GO:0022610 | +1.42 | 0.011 | +1.78 | 0.0519 |
| aromatic compound biosynthetic process | GO:0019438 | +1.44 | 0.011 | -0.98 | 1 |
| positive regulation of nitrogen compound metabolic process | GO:0051173 | +1.19 | 0.0119 | +1.23 | 0.553 |
| positive regulation of molecular function | GO:0044093 | +1.29 | 0.0131 | +1.48 | 0.146 |
| regulation of protein complex assembly | GO:0043254 | +1.57 | 0.0131 | +1.7 | 0.449 |
| cellular response to organonitrogen compound | GO:0071417 | +1.57 | 0.0134 | +1.94 | 0.151 |
| cellular response to nitrogen compound | GO:1901699 | +1.53 | 0.0134 | +1.74 | 0.311 |
| collagen fibril organization | GO:0030199 | +3 | 0.0134 | +2.97 | 0.752 |
| cation transport | GO:0006812 | +1.43 | 0.0135 | +1.94 | 0.0125 |
| response to oxygen-containing compound | GO:1901700 | +1.31 | 0.0136 | +1.41 | 0.435 |
| regulation of programmed cell death | GO:0043067 | +1.29 | 0.0136 | +1.31 | 0.682 |
| regulation of locomotion | GO:0040012 | +1.35 | 0.0142 | +1.58 | 0.137 |
| response to endogenous stimulus | GO:0009719 | +1.34 | 0.0142 | +1.36 | 0.739 |
| regulation of intracellular signal transduction | GO:1902531 | +1.28 | 0.0143 | +1.48 | 0.104 |
| positive regulation of organelle organization | GO:0010638 | +1.48 | 0.0144 | +1.43 | 0.772 |
| regulation of cellular biosynthetic process | GO:0031326 | +1.17 | 0.015 | +1.32 | 0.0693 |
| regulation of actin filament organization | GO:0110053 | +1.74 | 0.015 | +2.24 | 0.131 |
| cellular response to endogenous stimulus | GO:0071495 | +1.37 | 0.0152 | +1.47 | 0.503 |
| regulation of actin filament-based process | GO:0032970 | +1.58 | 0.0166 | +2.01 | 0.118 |
| pigment biosynthetic process | GO:0046148 | +3.04 | 0.0167 | -NA | 1 |
| positive regulation of cellular component movement | GO:0051272 | +1.47 | 0.0172 | +1.92 | 0.0508 |
| adaptive immune response | GO:0002250 | -0.55 | 0.018 | -0.15 | 0.0296 |
| regulation of biosynthetic process | GO:0009889 | +1.17 | 0.0181 | +1.33 | 0.0434 |
| regulation of apoptotic process | GO:0042981 | +1.28 | 0.0181 | +1.34 | 0.613 |
| biological regulation | GO:0065007 | +1.07 | 0.0185 | +1.18 | 0.000319 |
| immune response-regulating cell surface receptor signaling pathway | GO:0002768 | -0.47 | 0.0188 | -0.11 | 0.101 |
| ion transport | GO:0006811 | +1.32 | 0.0192 | +1.94 | 0.000336 |
| positive regulation of locomotion | GO:0040017 | +1.46 | 0.0192 | +1.88 | 0.0741 |
| immune response-activating cell surface receptor signaling pathway | GO:0002429 | -0.47 | 0.0197 | -0.11 | 0.101 |
| positive regulation of protein modification process | GO:0031401 | +1.31 | 0.0197 | +1.54 | 0.123 |
| divalent inorganic cation homeostasis | GO:0072507 | +1.5 | 0.0198 | +2.23 | 0.00665 |
| response to bacterium | GO:0009617 | -0.66 | 0.0198 | -0.51 | 0.356 |
| immune response | GO:0006955 | -0.74 | 0.0199 | -0.5 | 0.0539 |
| sphingolipid biosynthetic process | GO:0030148 | +2.57 | 0.0204 | -0.5 | 1 |
| nucleobase-containing small molecule metabolic process | GO:0055086 | +1.55 | 0.0209 | +1.09 | 1 |
| organophosphate biosynthetic process | GO:0090407 | +1.61 | 0.0216 | -0.78 | 1 |
| protein phosphorylation | GO:0006468 | +1.41 | 0.0216 | +1.12 | 1 |
| cellular divalent inorganic cation homeostasis | GO:0072503 | +1.52 | 0.0221 | +2.35 | 0.00413 |
| regulation of cellular localization | GO:0060341 | +1.38 | 0.0224 | +1.75 | 0.0619 |
| cofactor metabolic process | GO:0051186 | +1.59 | 0.0228 | -0.49 | 0.874 |
| endosomal transport | GO:0016197 | +1.84 | 0.0229 | +2.12 | 0.422 |
| regulation of cell adhesion | GO:0030155 | +1.42 | 0.0242 | +1.37 | 0.863 |
| mitochondrion organization | GO:0007005 | +1.58 | 0.0248 | +1.83 | 0.355 |
| purine-containing compound metabolic process | GO:0072521 | +1.65 | 0.0254 | +1.14 | 1 |
| vesicle budding from membrane | GO:0006900 | +2.76 | 0.0255 | +1.94 | 1 |
| cellular response to chemical stimulus | GO:0070887 | +1.21 | 0.0264 | +1.34 | 0.209 |
| cofactor biosynthetic process | GO:0051188 | +1.92 | 0.0267 | -NA | 0.502 |
| regulation of actin polymerization or depolymerization | GO:0008064 | +1.86 | 0.0268 | +1.94 | 0.692 |
| ceramide biosynthetic process | GO:0046513 | +2.85 | 0.0275 | -0.74 | 1 |
| metal ion transport | GO:0030001 | +1.46 | 0.0283 | +1.88 | 0.0815 |
| mRNA metabolic process | GO:0016071 | +1.47 | 0.0293 | +2.01 | 0.0429 |
| membrane lipid biosynthetic process | GO:0046467 | +2.18 | 0.0301 | -0.66 | 1 |
| regulation of actin filament polymerization | GO:0030833 | +1.91 | 0.0303 | +1.49 | 1 |
| organophosphate metabolic process | GO:0019637 | +1.4 | 0.0303 | +1.11 | 1 |
| regulation of protein catabolic process | GO:0042176 | +1.56 | 0.0304 | +1.83 | 0.354 |
| cell-cell adhesion | GO:0098609 | +1.56 | 0.0306 | +1.83 | 0.355 |
| calcium ion homeostasis | GO:0055074 | +1.5 | 0.031 | +2.07 | 0.0401 |
| glycosphingolipid metabolic process | GO:0006687 | +3.04 | 0.0311 | +2.67 | 0.89 |
| carboxylic acid catabolic process | GO:0046395 | +1.83 | 0.0314 | +1.49 | 1 |
| organic acid catabolic process | GO:0016054 | +1.83 | 0.0316 | +1.49 | 1 |
| monocarboxylic acid metabolic process | GO:0032787 | +1.5 | 0.0322 | +1.15 | 1 |
| cellular calcium ion homeostasis | GO:0006874 | +1.5 | 0.0324 | +2.15 | 0.0311 |
| cell motility | GO:0048870 | +1.34 | 0.0331 | +1.34 | 0.829 |
| localization of cell | GO:0051674 | +1.34 | 0.0333 | +1.34 | 0.829 |
| pigment metabolic process | GO:0042440 | +2.62 | 0.0345 | -0.61 | 1 |
| regulation of gene expression | GO:0010468 | +1.15 | 0.0354 | +1.32 | 0.0508 |
| rRNA transcription | GO:0009303 | +4.47 | 0.0368 | +4.19 | 0.844 |
| organelle localization | GO:0051640 | +1.5 | 0.0369 | +1.39 | 1 |
| cellular response to organic substance | GO:0071310 | +1.23 | 0.037 | +1.33 | 0.426 |
| regulation of actin filament length | GO:0030832 | +1.83 | 0.0382 | +1.91 | 0.812 |
| positive regulation of phosphate metabolic process | GO:0045937 | +1.3 | 0.0383 | +1.5 | 0.256 |
| positive regulation of phosphorus metabolic process | GO:0010562 | +1.3 | 0.0385 | +1.5 | 0.257 |
| movement of cell or subcellular component | GO:0006928 | +1.27 | 0.0385 | +1.37 | 0.491 |
| regulation of vasculature development | GO:1901342 | +1.59 | 0.0386 | +1.47 | 1 |
| inorganic ion transmembrane transport | GO:0098660 | +1.51 | 0.0388 | +1.83 | 0.288 |
| positive regulation of vasculature development | GO:1904018 | +1.78 | 0.0391 | -0.89 | 1 |
| regulation of response to stress | GO:0080134 | +1.27 | 0.0392 | +1.27 | 0.867 |
| positive regulation of cytoskeleton organization | GO:0051495 | +1.75 | 0.0394 | +2.18 | 0.325 |
| heme biosynthetic process | GO:0006783 | +3.98 | 0.0394 | -NA | 1 |
| leukocyte mediated immunity | GO:0002443 | -0.51 | 0.0397 | -NA | 0.0115 |
| endomembrane system organization | GO:0010256 | +1.56 | 0.0411 | +1.27 | 1 |
| fatty acid elongation | GO:0030497 | +4.88 | 0.0412 | -NA | 1 |
| negative regulation of cell death | GO:0060548 | +1.32 | 0.0413 | +1.47 | 0.403 |
| energy derivation by oxidation of organic compounds | GO:0015980 | +1.8 | 0.0414 | +2.44 | 0.168 |
| cellular metal ion homeostasis | GO:0006875 | +1.43 | 0.0418 | +2.16 | 0.006 |
| regulation of transferase activity | GO:0051338 | +1.35 | 0.0418 | +1.67 | 0.0982 |
| protein targeting | GO:0006605 | +1.75 | 0.0419 | +1.81 | 0.747 |
| response to organic substance | GO:0010033 | +1.19 | 0.0431 | +1.22 | 0.744 |
| phagocytosis | GO:0006909 | -0.49 | 0.0431 | -0.34 | 0.753 |
| circulatory system development | GO:0072359 | +1.34 | 0.0432 | +1.53 | 0.311 |
| monocarboxylic acid biosynthetic process | GO:0072330 | +1.95 | 0.0432 | +1.57 | 1 |
| collagen metabolic process | GO:0032963 | +2.62 | 0.0433 | +1.3 | 1 |
| regulation of angiogenesis | GO:0045765 | +1.62 | 0.0435 | +1.52 | 1 |
| actin filament-based process | GO:0030029 | +1.45 | 0.0436 | +1.84 | 0.123 |
| positive regulation of epithelial cell migration | GO:0010634 | +1.91 | 0.0436 | +2.67 | 0.171 |
| fatty acid biosynthetic process | GO:0006633 | +2.23 | 0.0439 | -0.77 | 1 |
| purine-containing compound biosynthetic process | GO:0072522 | +2 | 0.0447 | -0.83 | 1 |
| negative regulation of protein metabolic process | GO:0051248 | +1.31 | 0.0459 | +1.77 | 0.012 |
| regulation of biological process | GO:0050789 | +1.06 | 0.0467 | +1.18 | 0.00151 |
| lymphocyte mediated immunity | GO:0002449 | -0.5 | 0.048 | -NA | 0.0299 |
| heme metabolic process | GO:0042168 | +3.29 | 0.0488 | +1.19 | 1 |
| nucleobase-containing compound biosynthetic process | GO:0034654 | +1.4 | 0.0499 | +1.04 | 1 |
| cellular cation homeostasis | GO:0030003 | +1.39 | 0.0647 | +2.06 | 0.00835 |
| cytoskeleton organization | GO:0007010 | +1.3 | 0.07 | +1.87 | 0.00291 |
| positive regulation of cell communication | GO:0010647 | +1.22 | 0.0707 | +1.51 | 0.0498 |
| cellular chemical homeostasis | GO:0055082 | +1.36 | 0.0736 | +2.08 | 0.00268 |
| negative regulation of catabolic process | GO:0009895 | +1.58 | 0.0826 | +2.94 | 0.000943 |
| regulation of cell projection organization | GO:0031344 | +1.34 | 0.0832 | +2.35 | 2.45E-05 |
| metal ion homeostasis | GO:0055065 | +1.37 | 0.0904 | +2.03 | 0.0119 |
| regulation of multicellular organismal process | GO:0051239 | +1.15 | 0.103 | +1.48 | 0.000889 |
| negative regulation of cellular catabolic process | GO:0031330 | +1.62 | 0.106 | +2.93 | 0.00582 |
| regulation of membrane potential | GO:0042391 | +1.45 | 0.106 | +2.26 | 0.0143 |
| cellular ion homeostasis | GO:0006873 | +1.36 | 0.107 | +2.02 | 0.0126 |
| cellular homeostasis | GO:0019725 | +1.31 | 0.109 | +1.96 | 0.00403 |
| cation homeostasis | GO:0055080 | +1.34 | 0.113 | +1.93 | 0.0166 |
| RNA splicing | GO:0008380 | +1.53 | 0.119 | +2.69 | 0.00422 |
| ion homeostasis | GO:0050801 | +1.32 | 0.129 | +1.87 | 0.0212 |
| regulation of cellular process | GO:0050794 | +1.06 | 0.132 | +1.19 | 0.000823 |
| regulation of neurotransmitter levels | GO:0001505 | +1.47 | 0.14 | +2.49 | 0.00782 |
| regulation of plasma membrane bounded cell projection organization | GO:0120035 | +1.31 | 0.149 | +2.33 | 3.44E-05 |
| regulation of developmental process | GO:0050793 | +1.16 | 0.155 | +1.58 | 0.000239 |
| positive regulation of cell projection organization | GO:0031346 | +1.41 | 0.158 | +2.93 | 6.91E-06 |
| positive regulation of transport | GO:0051050 | +1.26 | 0.159 | +1.88 | 0.0019 |
| inorganic ion homeostasis | GO:0098771 | +1.32 | 0.177 | +1.9 | 0.0249 |
| inner mitochondrial membrane organization | GO:0007007 | +2.88 | 0.178 | +7.37 | 0.0299 |
| regulation of anatomical structure size | GO:0090066 | +1.36 | 0.18 | +2.19 | 0.00537 |
| RNA splicing, via transesterification reactions | GO:0000375 | +1.63 | 0.205 | +2.91 | 0.0241 |
| RNA splicing, via transesterification reactions with bulged adenosine as nucleophile | GO:0000377 | +1.63 | 0.206 | +2.91 | 0.0243 |
| mRNA splicing, via spliceosome | GO:0000398 | +1.63 | 0.206 | +2.91 | 0.0244 |
| mRNA processing | GO:0006397 | +1.42 | 0.214 | +2.2 | 0.0375 |
| gliogenesis | GO:0042063 | +1.55 | 0.228 | +3.18 | 0.00159 |
| neurogenesis | GO:0022008 | +1.19 | 0.232 | +2.06 | 3.17E-08 |
| myelination | GO:0042552 | +1.83 | 0.242 | +3.5 | 0.045 |
| regulation of transmembrane transport | GO:0034762 | +1.34 | 0.244 | +2.4 | 0.000448 |
| positive regulation of cellular component biogenesis | GO:0044089 | +1.36 | 0.244 | +2.23 | 0.00532 |
| negative regulation of protein catabolic process | GO:0042177 | +1.73 | 0.277 | +3.24 | 0.0477 |
| negative regulation of signal transduction | GO:0009968 | +1.21 | 0.351 | +1.63 | 0.0476 |
| negative regulation of cell communication | GO:0010648 | +1.2 | 0.366 | +1.7 | 0.0118 |
| response to inorganic substance | GO:0010035 | +1.36 | 0.372 | +2.25 | 0.0249 |
| regulation of cell morphogenesis | GO:0022604 | +1.31 | 0.391 | +2.32 | 0.0019 |
| negative regulation of signaling | GO:0023057 | +1.2 | 0.391 | +1.69 | 0.0119 |
| neurotransmitter transport | GO:0006836 | +1.57 | 0.451 | +3.16 | 0.0177 |
| regulation of secretion | GO:0051046 | +1.23 | 0.456 | +1.75 | 0.0428 |
| positive regulation of multicellular organismal process | GO:0051240 | +1.15 | 0.457 | +1.53 | 0.0183 |
| regulation of neuron projection development | GO:0010975 | +1.27 | 0.486 | +2.64 | 9.29E-06 |
| regulation of ion transport | GO:0043269 | +1.25 | 0.51 | +2.18 | 0.000693 |
| regulation of ion transmembrane transport | GO:0034765 | +1.3 | 0.544 | +2.2 | 0.0119 |
| positive regulation of neurogenesis | GO:0050769 | +1.26 | 0.589 | +2.44 | 0.000186 |
| anterograde trans-synaptic signaling | GO:0098916 | +1.33 | 0.622 | +2.32 | 0.0294 |
| chemical synaptic transmission | GO:0007268 | +1.33 | 0.622 | +2.32 | 0.0295 |
| cellular developmental process | GO:0048869 | +1.09 | 0.629 | +1.51 | 3.47E-05 |
| positive regulation of nervous system development | GO:0051962 | +1.23 | 0.667 | +2.35 | 0.000204 |
| positive regulation of cell development | GO:0010720 | +1.23 | 0.676 | +2.2 | 0.00123 |
| generation of neurons | GO:0048699 | +1.14 | 0.698 | +2.04 | 2.10E-07 |
| regulation of neurogenesis | GO:0050767 | +1.19 | 0.701 | +2.28 | 5.97E-06 |
| positive regulation of neuron projection development | GO:0010976 | +1.32 | 0.702 | +3.19 | 1.23E-05 |
| central nervous system development | GO:0007417 | +1.2 | 0.709 | +1.89 | 0.0125 |
| regulation of neuron differentiation | GO:0045664 | +1.2 | 0.733 | +2.52 | 1.61E-06 |
| regulation of cell differentiation | GO:0045595 | +1.13 | 0.743 | +1.76 | 9.53E-05 |
| cell differentiation | GO:0030154 | +1.09 | 0.744 | +1.53 | 1.61E-05 |
| regulation of cell development | GO:0060284 | +1.17 | 0.751 | +2.06 | 7.09E-05 |
| positive regulation of neuron differentiation | GO:0045666 | +1.26 | 0.757 | +2.89 | 1.20E-05 |
| trans-synaptic signaling | GO:0099537 | +1.29 | 0.757 | +2.31 | 0.024 |
| cristae formation | GO:0042407 | +2.71 | 0.769 | +12.72 | 0.0139 |
| regulation of nervous system development | GO:0051960 | +1.17 | 0.771 | +2.13 | 1.61E-05 |
| protein acylation | GO:0043543 | +1.43 | 0.803 | +3.02 | 0.0363 |
| memory | GO:0007613 | +1.46 | 0.814 | +4.15 | 0.000608 |
| regulation of multicellular organismal development | GO:2000026 | +1.11 | 0.814 | +1.59 | 0.002 |
| microtubule cytoskeleton organization | GO:0000226 | +1.24 | 0.829 | +2.17 | 0.0237 |
| modulation of chemical synaptic transmission | GO:0050804 | +1.23 | 0.848 | +2.58 | 0.000151 |
| regulation of trans-synaptic signaling | GO:0099177 | +1.23 | 0.849 | +2.57 | 0.000151 |
| learning or memory | GO:0007611 | +1.3 | 0.872 | +2.93 | 0.00143 |
| cell development | GO:0048468 | +1.11 | 0.914 | +1.61 | 0.00661 |
| amino acid transport | GO:0006865 | +1.46 | 0.986 | +3.6 | 0.0386 |
| behavior | GO:0007610 | +1.12 | 1 | +2.39 | 6.18E-05 |
| cognition | GO:0050890 | +1.23 | 1 | +2.95 | 0.000433 |
| long-term memory | GO:0007616 | +1.56 | 1 | +8.22 | 0.0011 |
| exploration behavior | GO:0035640 | +1.22 | 1 | +9.19 | 0.00164 |
| regulation of growth | GO:0040008 | +1.16 | 1 | +2.14 | 0.00167 |
| regulation of microtubule-based process | GO:0032886 | +1.26 | 1 | +2.96 | 0.00542 |
| regulation of cell morphogenesis involved in differentiation | GO:0010769 | +1.13 | 1 | +2.56 | 0.00567 |
| regulation of cell growth | GO:0001558 | +1.17 | 1 | +2.36 | 0.00754 |
| positive regulation of cell differentiation | GO:0045597 | +1.13 | 1 | +1.78 | 0.00828 |
| neuron projection development | GO:0031175 | +1.05 | 1 | +2.02 | 0.00874 |
| neuron development | GO:0048666 | +1.04 | 1 | +1.91 | 0.00997 |
| associative learning | GO:0008306 | +1.09 | 1 | +4.11 | 0.0101 |
| neuron differentiation | GO:0030182 | -0.98 | 1 | +1.79 | 0.0102 |
| positive regulation of axonogenesis | GO:0050772 | +1.37 | 1 | +4.07 | 0.0107 |
| action potential | GO:0001508 | +1.28 | 1 | +4.4 | 0.0109 |
| acidic amino acid transport | GO:0015800 | +1.84 | 1 | +7.56 | 0.0111 |
| regulation of synaptic plasticity | GO:0048167 | +1.01 | 1 | +2.94 | 0.0111 |
| regulation of microtubule cytoskeleton organization | GO:0070507 | +1.27 | 1 | +2.97 | 0.0141 |
| regulation of axonogenesis | GO:0050770 | +1.14 | 1 | +2.93 | 0.0163 |
| regulation of cation transmembrane transport | GO:1904062 | +1.23 | 1 | +2.39 | 0.0176 |
| learning | GO:0007612 | -0.98 | 1 | +3.12 | 0.0194 |
| locomotory exploration behavior | GO:0035641 | +1.42 | 1 | +11.13 | 0.0211 |
| regulation of long-term synaptic potentiation | GO:1900271 | +1.02 | 1 | +5.48 | 0.0215 |
| regulation of transporter activity | GO:0032409 | +1.25 | 1 | +2.55 | 0.0228 |
| locomotory behavior | GO:0007626 | +1.04 | 1 | +2.67 | 0.0261 |
| positive regulation of synaptic transmission | GO:0050806 | +1.05 | 1 | +2.95 | 0.0297 |
| response to zinc ion | GO:0010043 | +1.01 | 1 | +7.12 | 0.0342 |
| mating | GO:0007618 | +1.18 | 1 | +5.54 | 0.0421 |
| positive regulation of cell morphogenesis involved in differentiation | GO:0010770 | +1.25 | 1 | +2.83 | 0.0425 |
| multi-organism behavior | GO:0051705 | -0.9 | 1 | +4.22 | 0.0426 |
| histone modification | GO:0016570 | +1.17 | 1 | +2.35 | 0.0435 |
| negative regulation of RNA catabolic process | GO:1902369 | +1.62 | 1 | +4.67 | 0.0455 |
| regulation of tube diameter | GO:0035296 | +1.24 | 1 | +2.9 | 0.048 |
| regulation of blood vessel diameter | GO:0097746 | +1.24 | 1 | +2.9 | 0.0483 |
| histone lysine methylation | GO:0034968 | -0.86 | 1 | +4.6 | 0.0485 |
| cytoplasmic microtubule organization | GO:0031122 | +1.35 | 1 | +4.6 | 0.0487 |
| regulation of tube size | GO:0035150 | +1.23 | 1 | +2.88 | 0.0499 |
